# Population pharmacokinetic model for oral ORIN1001 in Chinese patients with advanced solid tumors

**DOI:** 10.3389/fphar.2024.1322557

**Published:** 2024-03-04

**Authors:** Xiaoqing Li, Yunhai Bo, Qingping Zeng, Lei Diao, Stephanie Greene, John Patterson, Lu Liu, Fen Yang

**Affiliations:** ^1^ Key Laboratory of Carcinogenesis and Translational Research (Ministry of Education), National Drug Clinical Trial Center, Peking University Cancer Hospital and Institute, Beijing, China; ^2^ Fosun Orinove, Inc., Suzhou, China; ^3^ Shanghai Fosun Pharmaceutical Development Co., Ltd., Shanghai, China

**Keywords:** population pharmacokinetic model, ORIN1001, model construction, model evaluation, model simulation

## Abstract

**Background:** ORIN1001, a first-in-class oral IRE1-α endoribonuclease inhibitor to block the activation of XBP1, is currently in clinical development for inhibiting tumor growth and enhancing the effect of chemical or targeted therapy. Early establishment of a population pharmacokinetic (PopPK) model could characterize the pharmacokinetics (PK) of ORIN1001 and evaluate the effects of individual-specific factors on PK, which will facilitate the future development of this investigational drug.

**Methods:** Non-linear mixed effect model was constructed by Phoenix NLME software, utilizing the information from Chinese patients with advanced solid tumors in a phase I clinical trial (Register No. NCT05154201). Statistically significant PK covariates were screened out by a stepwise process. The final model, after validating by the goodness-of-fit plots, non-parametric bootstrap, visual predictive check and test of normalized prediction distribution errors, was further applied to simulate and evaluate the impact of covariates on ORIN1001 exposure at steady state up to 900 mg per day as a single agent.

**Results:** A two-compartment model with first-order absorption (with lag-time)/elimination was selected as the best structural model. Total bilirubin (TBIL) and lean body weight (LBW) were considered as the statistically significant covariates on clearance (CL/F) of ORIN1001. They were also confirmed to exert clinically significant effects on ORIN1001 steady-state exposure after model simulation. The necessity of dose adjustments based on these two covariates remains to be validated in a larger population.

**Conclusion:** The first PopPK model of ORIN1001 was successfully constructed, which may provide some important references for future research.

## 1 Introduction

Unfolded protein response (UPR), which is triggered by the accumulation of misfolded proteins exceeding the tolerable threshold of endoplasmic reticulum (ER), repairs the ER protein folding ability by enhancing the transcription and translation of chaperones and protein degradation factors (Wang and Kaufman, 2014). It has been confirmed as a major survival pathway of eukaryotes under stress (Ron and Walter, 2007; Hetz and Glimcher, 2009; Walter and Ron, 2011), and also a supporter for the growth of tumor cells, matrix and vascular systems through synthesizing anti-apoptotic proteins and secreting a variety of cytokines (Wang and Kaufman, 2014; Papaioannou and Chevet, 2018; Song et al., 2018). UPR promotes tumorigenicity, metastasis, drug resistance and adaptation to adverse micro-environment (nutrient deprivation, oxygen restriction, high metabolic demand and oxidative stress as examples) (Cubillos-Ruiz et al., 2017) of some solid tumors, such as breast, pancreatic, lung and skin cancers (Wang and Kaufman, 2016; Avril et al., 2017; Cubillos-Ruiz et al., 2017; Logue et al., 2018).

IRE1-α, a unique trans-membrane kinase-endoribonuclease signaling molecule of ER, is the most conservative and prominent enzyme in UPR (Lee et al., 2008; Lu et al., 2014). The transcription factor XBP1 could be activated by IRE1-α endoribonuclease (RNase), and then send signals including protein folding, glycosylation, quality control and lipid synthesis (Yoshida et al., 2001; Chen et al., 2020). Therefore, the small molecule inhibitor targeting IRE1-α and XBP1 to suppress UPR is considered as an important agent for tumor treatment and recurrence (Xie et al., 2018; Zhao et al., 2018).

ORIN1001 is a first-in-class IRE1-α RNase inhibitor to block the activation of XBP1 (Gabrail et al., 2021). In preclinical studies, ORIN1001 exhibited moderate anti-tumor efficacy when applied alone and also synergistic activity when used with standard therapeutic agents (such as docetaxel or paclitaxel). Accordingly, ORIN1001 is being studied for single-use or combined-use with standard treatment in clinical trials in order to explore its safety and effectiveness.

This study aims to establish a precise population pharmacokinetic (PopPK) model of ORIN1001 in order to evaluate the effects of internal and external factors on its pharmacokinetics (PK), based on existing information from a phase I clinical trial (single-use). The final developed PopPK model was used to not only characterize the PK of ORIN1001 but also explore some covariates with clinical significance on drug steady-state exposure after model simulation. This PopPK model will provide an important reference for subsequent clinical research of ORIN1001.

## 2 Methods

### 2.1 Clinical information

The dataset used for PopPK modeling was from the phase I clinical trial (Register No. NCT05154201) of ORIN1001, a basket clinical trial currently underway in Chinese patients with advanced solid tumors. This trial, approved by the Ethics Committee of Peking University Cancer Hospital and in accordance with the Declaration of Helsinki, was an open-label, dose-increasing and dose-extending study. All recruited subjects had signed informed consent forms. They were divided into seven groups and received ORIN1001 tablets orally once a day at different dose levels (100, 200, 300, 400, 500, 650 and 900 mg). Throughout the research process, they were observed for 4 days following a single dose (single-dose period) and then for a cycle of 21 days after being administered daily (multiple-dose period). Sparse blood samples were mainly collected before and after the administration at 1, 2, 4, 6, 8, 12, 24, 48, 72 and 96 h in the single-dose period, and at 1, 2, 4, 6, 8, 12 and 24 h on day 21 in the multiple-dose period.

### 2.1 Analytical methods

The concentration of ORIN1001 in plasma was determined by a validated liquid chromatography-tandem mass spectrometry (LC-MS/MS) method with ORIN1001-d_8_ applied as the internal standard. After protein precipitated using methanol, the samples were chromatographed on the Waters Xbridge C18 column (2.1*50 mm, 3.5 μm particle size) with a column temperature at 40°C. The mobile phase A was water (containing 0.1% formic acid), and the mobile phase B was the combination of acetonitrile and methanol (50:50, v/v, containing 0.1% formic acid). The gradient was performed with the total flow at 0.6 mL/min as follows: 0–0.3 min 25%–25% B, 0.3–1.5 min 25%–55% B, 1.5–1.6 min 55%–95% B, 1.6–2.5 min 95%–95% B, followed by the re-equilibration for 2.0 min before the next injection. Mass spectrometric analysis was conducted on Sciex API 5500 equipped with a positive electrospray ionization source (ESI+). The settings for ESI source were as follows: IonSpray voltage, 5,500 V; Source temperature, 500°C; Collision gas, 9 units; Curtain gas, 40 psi; Nebulizing gas, 50 psi; Auxiliary gas, 60 psi; Entrance potential, 10 V. Multiple reaction monitoring (MRM) transitions and related collision energy were m/z 362.2→247.0 (30 eV) for ORIN1001 and m/z 370.3→247.1 (30 eV) for ORIN1001-d_8_. The linear range of ORIN1001 in plasma was 50–20,000 ng/mL. Accuracy and precision were both within the acceptable range of bioanalytical assay validation criteria (e.g., ±15%) issued by the Food and Drug Administration.

### 2.3 PK study

The PK parameters of ORIN1001 were calculated by the non-compartmental analysis (NCA) using Phoenix (RRID:SCR_003163) WinNonlin software (version 8.3, Pharsight Corporation, CA, United States), including clearance rate (CL/F), apparent distribution volume (V_z_/F), elimination half-life (t_1/2_), the area under the concentration-time curve from zero to the last time (AUC_0-t_) and from zero to infinity (AUC_0-inf_). Other parameters were acquired directly from the observations, for instance, maximum observed plasma concentration (C_max_) and time to C_max_ (T_max_).

The dose proportionality of ORIN1001 was assessed in both single- and multiple-dose. The calibration curve of ORIN1001 ranging from 100 to 900 mg was constructed by regression of the mean exposure (Y) of all subjects *versus* the dose (X). The linearity was assessed in terms of the correlation coefficient (R) which should be ≥0.99.

### 2.4 PopPK analysis

Non-linear mixed effect model was constructed using Phoenix (RRID:SCR_003163) NLME (Version 8.3, Pharsight Corporation, CA, United States), in which the first-order conditional estimation-extended least-squares (FOCE-ELS) method was used for model parameter estimation and test. R studio was applied for statistical analysis and data visualization during the modeling process (Version 4.2.0, R Project for Statistical Computing, RRID:SCR_001905, http://www.r-project.org/).

#### 2.4.1 Basic model

Based on the results of the dose proportionality, one-, two- and three-compartment models with zero-/first-order absorption and first-order elimination were attempted as the structural model, respectively. The parameter lag-time (T_lag_) was tried to add in the absorption phase to explain the delay between administration and absorption. Inter-individual variability (η, eta, IIV) of the model parameters was described as an exponential model in Eq. (1):

$$P_{ij}={TVP}_{i}*e^{\eta_{ij}}$$ (1)

where TVP_i_ represented the typical estimation of the *i*th PopPK parameter, P_ij_ and η_ij_ were the estimation and IIV of the *i*th parameter for the *j*th subject, respectively.

Intra-individual variability (ε, epsilon, residual variability), was initially estimated by an additive, proportional or additive plus proportional error model. All random effects followed the normal distribution, including IIV with a mean of zero and a variance of ω^2^ (omega), as well as the intra-individual variability with a mean of 0 and a variance of σ^2^ (sigma).

#### 2.4.2 Covariate model

The influence of continuous covariates on PK parameters was modeled using the power function (Eq. (2)), with normalization by the population median of the study cohort:

$${Effect}_{i}={\left(\frac{{Cov}_{ij}}{{Cov}_{median}}\right)}^{\theta_{{Cov}_{i}}}$$ (2)

where Effect_i_ represented the influence of the *i*th covariate on specific PK parameters, Cov_ij_ was the actual covariate value of the *i*th covariate in the *j*th subject, Cov_median_ was the median of the covariate in study crowd, and θ_covi_ was regarded as the fixed effects on PK parameters from covariate i.

The detailed information of some continuous covariates was directly gained from the computer-based medical record of patients, including age, height, weight, blood urea nitrogen (BUN), blood creatinine (Cr), alkaline phosphatase (ALP), lactate dehydrogenase (LDH), total bilirubin (TBIL), direct bilirubin (DBIL), indirect bilirubin (IBIL), aspartate aminotransferase (AST) and alanine aminotransferase (AST). Derived covariates such as body mass index (BMI) (Keys et al., 1972), body surface area (BSA) (Stevenson, 1937), lean body weight (LBW) (Janmahasatian et al., 2005), ideal body weight (IBW) (Robinson et al., 1983), creatinine clearance rate (CLcr) (Cockcroft and Gault, 1976), the percentage of body fat (BF%) and adjusted CLcr (adj CLcr, calculated using adjusted weight for BMI>25 kg/m^2^) (Winter et al., 2012), were calculated for fully utilizing covariate information.

In order to avoid covariate collinearity, the Pearson test was applied for correlation analysis between two covariates before the covariate model establishment. Only one of them could be selected by the univariate analysis process when the correlation coefficient was larger than 0.5 (*p* < 0.05). Then this covariate would be considered as the candidate covariate in the subsequent stepwise approach, which is implemented to search statistically significant variables. In forward addition, one covariate would be remained if the decline in objective function value (OFV) was larger than 3.84 (*p* < 0.05, df = 1) after adding it. Then, in backward addition, one covariate would be kicked out when the increase in OFV was larger than 6.64 (*p* < 0.01, df = 1) after deleting it. Moreover, a non-diagonal variance-covariance matrix for IIVs was also assessed to improve the PopPK model fitting.

#### 2.4.3 Model superiority

At every stage of model establishment, model superiority comparisons were performed in order to obtain the best final model. Except for better examination of diagnostic plots, the reduction value of indicators (for example, the OFV, Akaike Information Criteria (AIC) and Bayesian information criteria (BIC)) exceeding 3.84 (*p* < 0.05, df = 1) also implied the superiority of PopPK model.

### 2.5 Model evaluation

The precision and accuracy (expressed as residual standard error, RSE%) of final PopPK parameter estimations were evaluated to illustrate the model fitness. Goodness of fit (GOF) plots, visual predictive check (VPC), non-parametric bootstrap and test of normalized distribution errors (NPDE) were all crucial validation methods for demonstrating model reliability and robustness. GOF plots of ORIN1001 concentration in plasma included the scatterplots of (i) the observation value (DV) *versus* (vs.) population predicted value (PRED), (ii) the DV vs. individual predicted value (IPRED), (iii) the conditional weighted residuals (CWRES) vs. PRED and (iv) CWRES vs. time. VPC, simulated by the Monte Carlo technique, was calculated at 1,000 replicates per time. Subsequently, VPC simulations were compared with observations in the fifth, 50th and 95th percentiles to evaluate the predictive ability of the PopPK model. During the bootstrap screening process, estimated PK parameters and their 95% confidence interval (CI) were figured out using the virtual population (randomly sampling 1,000 times from the original datasets), and then compared with the results of the final model. NPDE values, simulated 1,000 runs of the observations, were considered to follow the standard normal distribution if the PopPK model was sufficiently superior.

### 2.6 Model simulation

In order to explore the impact of statistically significant covariates on ORIN1001 exposure at steady state (minimum concentration, C_min,ss_; maximum concentration, C_max,ss_; area under concentration-time curve, AUC_ss_), simulations (n = 500 in every scenario) were conducted using final PopPK model in the virtual patients, whose covariates are defined as median (reference patients), 5th and 95th percentiles of covariate distribution (collectively considered as simulated patients). If the fold change of ORIN1001 steady-state exposure in simulated patients was within the range of 80%–125% relative to the reference patients after 900 mg dosing, inspected covariates were considered to have no clinical significance.

## 3 Results

### 3.1 Modeling dataset and PK parameters

**FIGURE 1:**
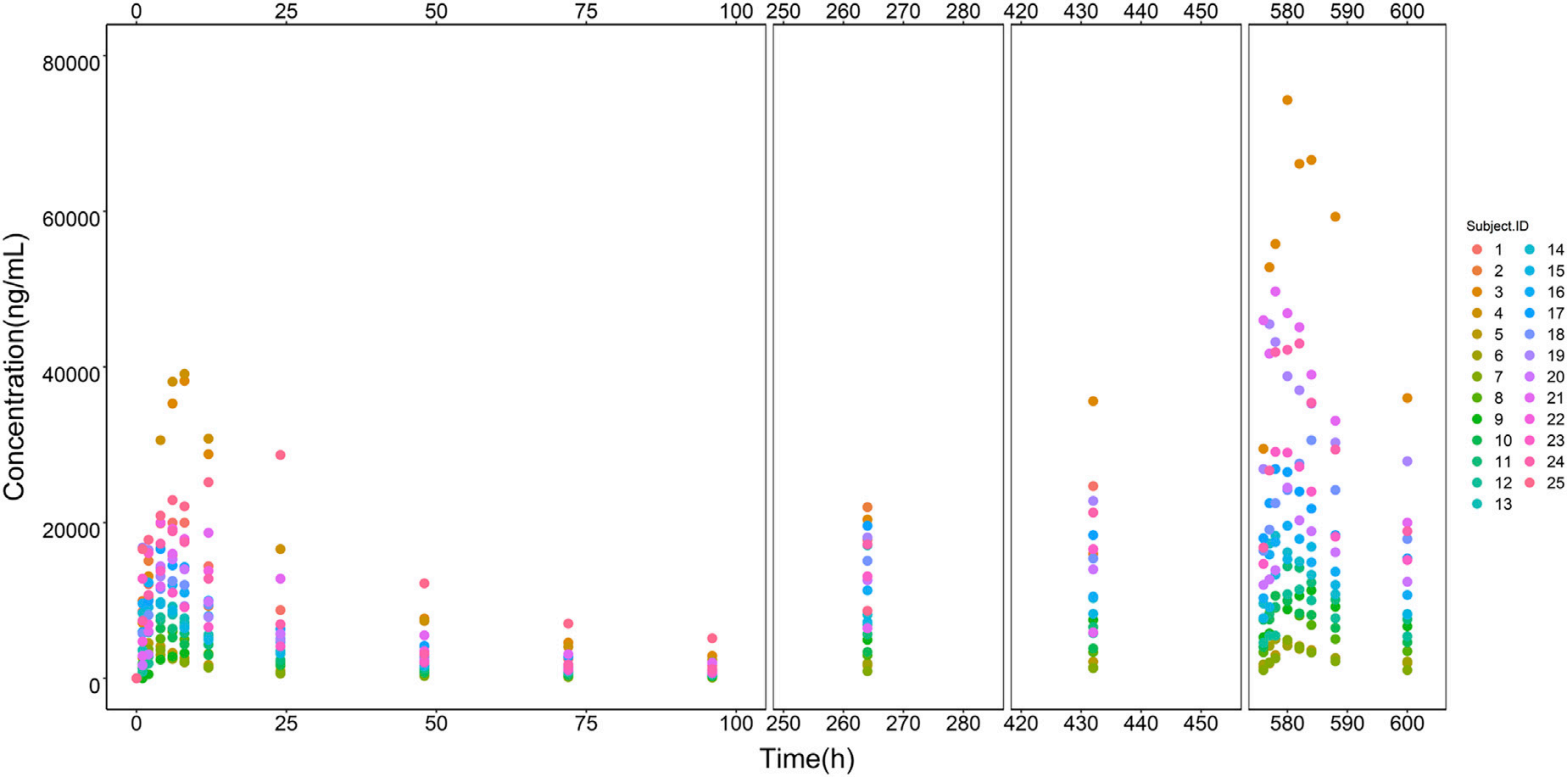
ORIN1001 concentration *versus* time points from all recruited subjects. The legend on the right shows the identification (ID) of each subject.

Modeling dataset was composed of 471 plasma drug concentrations (Figure 1) and clinical characteristics (Table 1) from 25 subjects. Corresponding PK parameters are calculated and displayed in Table 2. In the dose proportionality assessment (Supplementary Figure S1), the R values were 0.99 _(Cmax-single dose)_, 0.99 _(AUC0-t-single dose)_, 0.99 _(Cmax-multiple dose)_, and 0.97 _(AUC0-t-multiple dose)_, indicating the exposures of ORIN1001 were basically proportional to the dose. The dose-normalized plasma concentrations in the multiple-dose stage are shown in Supplementary Figure S2.

**TABLE 1:** Characteristics of baseline demographic and laboratory examinations^a^.

Characteristic	Median (IQR)	Range
Numbers of patients	25	
Numbers of concentrations	471	
Sex, male/female	13 (52%)/12 (48%)	
Age, years (*n* = 25)	57 (50.50–64)	37–72
Height, cm (*n* = 25)	165 (155.50–170.50)	150–178
Weight, kg (*n* = 25)	64 (57–70)	42–80
BMI, kg/m^2 (*n* = 25)	23.51 (20.83–26.21)	18.67–28.72
BSA, m^2 (*n* = 25)	1.66 (1.56–1.77)	1.30–1.95
LBW, kg (*n* = 25)	45.13 (38.09–53.73)	30–60.81
BF% (*n* = 25)	25.20 (20.38–36.97)	13.47–41.28
IBW, kg (*n* = 25)	58.70 (50.68–66.39)	47.11–73.18
Adj weight, kg (*n* = 25)	59.70 (55.77–66.76)	45.07–75.37
BUN, mmol/L (*n* = 25)	8.94 (7.12–11.09)	3.70–16.28
LDH, IU/L (*n* = 25)	214 (180.50–279)	98–1,268
Cr, μmol/L (*n* = 25)	58 (50.45–70)	31–89
CLcr, mg/dL (*n* = 25)	103.54 (81.71–117.87)	54.55–168.39
Adj CLcr, mg/dL (*n* = 25)	92.88 (80.31–109.23)	44.26–159.86
TBIL, μmol/L (*n* = 25)	10.30 (8.35–13.35)	4.80–23.20
DBIL, μmol/L (*n* = 25)	3 (2.15–4.90)	1.30–7.40
IBIL, μmol/L (*n* = 25)	7.9 (6.10–9.30)	2.40–26.90
ALT, IU/L (*n* = 25)	18.7 (10.75–25.55)	3–37
AST, IU/L (*n* = 25)	22 (16.50–28.50)	10–77
ALP, IU/L (*n* = 25)	85 (58–147.45)	31–304

^a^ IQR, interquartile range; BMI, body mass index; BSA, body surface area; LBW, lean body weight; BF%, body fat percentage; IBW, ideal body weight; Adj weight, adjusted weight; BUN, blood urea nitrogen; LDH, lactate dehydrogenase; Cr, serum creatinine; CLcr, endogenous creatinine clearance rate; Adj CLcr, adjusted endogenous creatinine clearance rate; TBIL, total bilirubin; DBIL, direct bilirubin; IBIL, indirect bilirubin, ALT, baseline alanine aminotransferase; AST, baseline aspartate transaminase; ALP, alkaline phosphatase.

**TABLE 2:** Summary of the ORIN1001 pharmacokinetic parameters calculated using the non-compartmental analysis (NCA).

	Dose (mg)	AUC_0-t_ ^a^ ^,^ ^b^ (ng·h/mL)	AUC_0-inf_ ^a^ (ng·h/mL)	C_max_ ^a^ (ng/mL)	T_max_ ^c^ (h)	t_1/2_ ^a^ (h)	V_z_/F^a^ (L)	CL/F^a^ (L/h)
**Single-dose**	100 (*n* = 3)	68788.93 (11862.47)	75962.73 (13066.34)	3820 (663.67)	2 (2,2)	33.26 (6.66)	64.02 (14.63)	1.34 (0.22)
	200 (*n* = 3)	131728.83 (7053.45)	145145.68 (10518.25)	5590 (1,092.34)	7.33 (4,12)	32.01 (1.36)	63.74 (2.71)	1.38 (0.10)
	300 (*n* = 4)	228262.75 (40115.46)	255482.73 (43998.63)	9360 (1947.67)	4 (2,6)	32.03 (2.65)	56.10 (14.76)	1.20 (0.22)
	400 (*n* = 3)	368183.33 (134885.27)	429240.23 (187717.63)	13446.67 (3589.78)	4 (4,4)	33.68 (8.52)	48.56 (14.41)	1.05 (0.43)
	500 (*n* = 3)	378935.33 (11038.56)	423552.73 (9794.66)	14866.67 (2182.51)	4.33 (1,6)	33.46 (5.25)	57.01 (9.18)	1.18 (0.03)
	650 (*n* = 5)	522604.40 (160299.01)	577313.57 (191065.33)	18300 (1826.20)	5.60 (2,12)	29.74 (4.81)	51.51 (14.21)	1.24 (0.43)
	900 (*n* = 4)	1043135 (389883.52)	1188790.39 (477250.87)	31225 (9468.32)	11.50 (6,24)	33.90 (3.82)	43.46 (23.54)	0.90 (0.48)
**Multiple- dose**	100 (*n* = 3)	66065 (7377.35)		4846.67 (226.79)	3.33 (2,4)	16.05 (5.98)	20.85 (1.68)	0.99 (0.35)
	200 (*n* = 3)	171946.67 (33840.18)		10633.33 (650.64)	4.67 (2,8)	20.19 (4.41)	18.65 (2.44)	0.67 (0.23)
	300 (*n* = 3)	239481.67 (52023.15)		14700 (3459.77)	4 (2,6)	21.25 (2.61)	20.64 (3.97)	0.68 (0.19)
	400 (*n* = 3)	371281.67 (95066.09)		21466.67 (4781.56)	4 (2,6)	25.53 (8.60)	18.76 (4.12)	0.55 (0.23)
	500 (*n* = 3)	579450 (201502.75)		33533.33 (10802.93)	4.33 (1,8)	31.04 (9.63)	16.37 (4.96)	0.40 (0.19)
	650 (*n* = 2)	661975		39400	2 (2,2)	18.91	16.29	0.59
	900 (*n* = 2)	1022700		58650	5 (4,6)	17.81	14.73	0.57

^a^ These data are displayed as mean (SD).

^b^ t means the time points at 100 h after single-dose and 24 h after multiple-dose on day 21.

^c^ T_max_ is displayed as median (minimum, maximum).

### 3.2 Model construction

**FIGURE 2:**
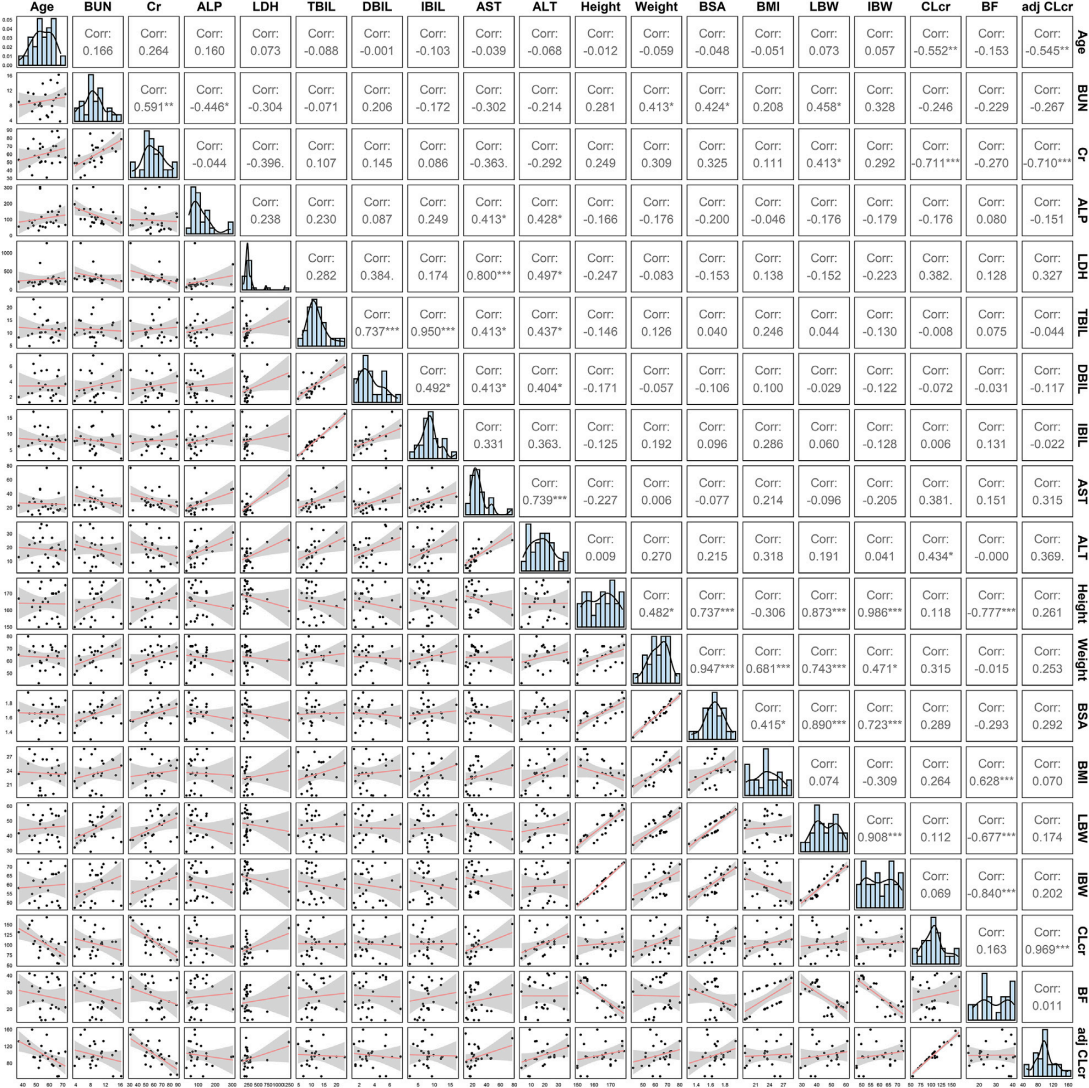
The covariate distribution and Pearson correlation between every two continuous covariates.

A two-compartment model with first-order absorption (with T_lag_)/elimination was selected as the best structural model. Exponential model and proportional model were applied to describe the inter- and intra-individual variability, respectively. In the establishment stage of the covariate model, Pearson correlation coefficient between every two continuous variables was evaluated (Figure 2). Remained covariates were then entered into the stepwise screening process, including BUN, ALP, LDH, TBIL, AST, BMI, LBW and adj CLcr. The results of stepwise procedures after forward and backward selection are presented in Table 3. Detailed stepwise screening processes are shown in the Supplementary Material. The decrease in OFV by more than 3.84, observed upon incorporating covariate effects, signified the statistical significance of TBIL, LBW, and LDH as covariates in this PopPK model. The computational formulas of CL/F in the final model are shown below in Eq. (3):

CLFLh=1.07*TBIL10.30−0.46*LBW45.131.11 (3)

where 1.07 L/h is the typical value of CL/F, as well as 10.3 μmol/L and 45.13 kg is the median of the covariate TBIL and LBW, respectively. The estimated effect coefficients such as −0.46 and 1.11, represent their covariate effect on PK parameters. The computational formulas of CL_2_/F, V/F and V_2_/F in the final model are shown in Supplementary Equations S1–S3.

**TABLE 3:** Results of stepwise procedure including forward inclusion and backward elimination^a^.

Step	Covariate screening	OFV	ΔOFV	*p*-value	Comments
1	None	7857.55			Base model
Forward inclusion					
2	CL_2_/F-BUN	7845.80	−11.75	<0.001	
3	CL/F-TBIL CL_2_/F-BUN	7840.51	−5.29	<0.05	
4	CL/F-TBIL-LBW CL_2_/F-BUN	7835.12	−6.39	<0.05	
5	CL/F-TBIL-LBW CL_2_/F-BUN V_2_/F-LDH	7829.18	−5.94	<0.05	
6	CL/F-TBIL-LBW CL_2_/F-BUN-LDH V_2_/F-LDH	7822.94	−6.24	<0.05	
7	CL/F-TBIL-LBW CL_2_/F-BUN-LDH-LBW V_2_/F-LDH	7818.38	−4.56	<0.05	Full model
Backward elimination					
8	CL/F-TBIL-LBW CL_2_/F-LDH-LBW V_2_/F-LDH	7822.57	4.19	>0.01	
9	CL/F-TBIL-LBW CL_2_/F-LBW V_2_/F-LDH	7828.45	5.88	>0.01	Final model

^a^ ΔOFV, the change value of OFV.

### 3.3 Model validation

**FIGURE 3:**
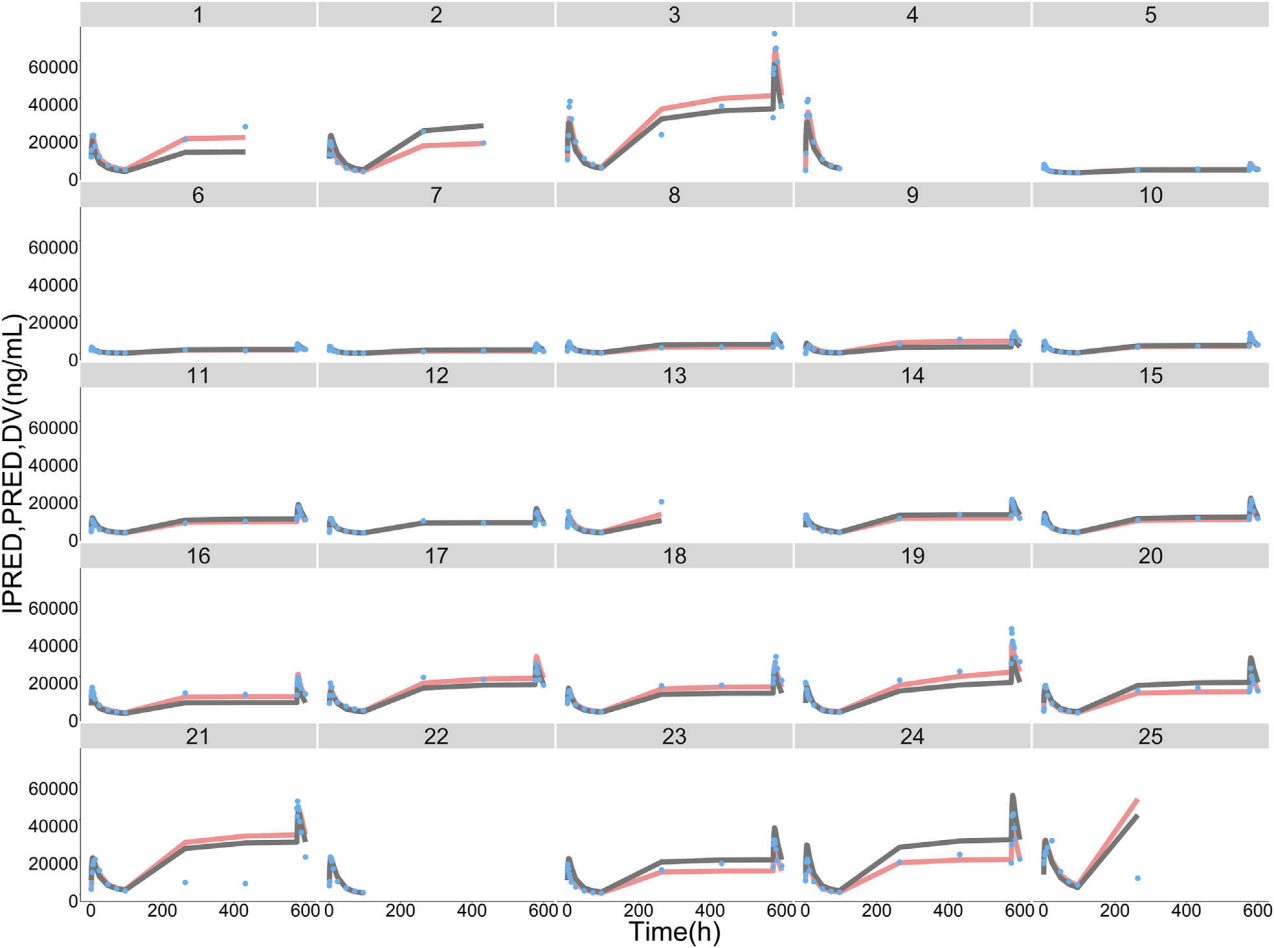
ORIN1001 concentration observations (DV, sky blue dots), population predictions (PRED, dark grey lines) and individual predictions (IPRED, pink lines) of the final population pharmacokinetic model. The numbers on the top of facets represent the corresponding subject identification.

Typical values, inter-individual variability and residual variability of PK parameters were estimated in the base model (Supplementary Table S1) and final model with RSE% less than 30% (Table 4). Furthermore, it is displayed in Figure 3 that the individual simulated concentration-time curves of the final model could match all the observations well.

**TABLE 4:** Estimation of pharmacokinetics parameters in the best final model and bootstrap procedure^a^.

	Final model		Bootstrap	
Parameters	Estimate (%RSE)	95% CI	Median (%RSE)	95% CI
K_a_ (1/h)	0.58 (17.12%)	0.39–0.78	0.61 (18.36%)	0.43–0.86
T_lag_	0.35 (19.63%)	0.22–0.49	0.34 (28.36%)	0.16–0.53
V/F (L)	26.21 (5.39%)	23.44–28.99	26.29 (6.08%)	24.07–32.29
V_2_/F (L)	26.60 (9.72%)	21.52–31.68	26.91 (13.41%)	21.03–34.73
CL/F (L/h)	1.07 (5.31%)	0.95–11.77	1.06 (5.89%)	0.94–1.18
CL_2_/F (L/h)	0.75 (12.57%)	0.57–0.94	0.76 (14.44%)	0.57–1.00
TBIL on CL/F	−0.46 (−22.51%)	−0.66 to −0.26	−0.46 (−42.27%)	−0.83 to −0.08
LBW on CL/F	1.11 (18.00%)	0.72–1.51	1.04 (29.76%)	0.38–1.63
LDH on V_2_/F	0.99 (19.33%)	0.61–1.36	0.94 (36.26%)	0.25–1.57
LBW on CL_2_/F	2.21 (19.30%)	1.37–3.05	2.10 (31.84%)	0.72–3.33
Inter-individual variability				
ω^2^ V/F	0.049 (28.32%)	0.022–0.076	0.045 (50.81%)	0.013–0.103
ω^2^ CL/F	0.067 (11.69%)	0.052–0.082	0.059 (22.25%)	0.037–0.085
ω^2^ K_a_	0.534 (26.66%)	0.255–0.814	0.540 (41.54%)	0.203–1.055
ω^2^ T_lag_	0.438 (19.33%)	0.272–0.604	0.505 (67.28%)	0.093–1.485
Residual variability (σ)				
stdev0	0.197 (7.48%)	0.168–0.226	0.195 (7.46%)	0.168–0.225

^a^ %RSE, the percentage of the relative standard error; CI, confidence interval; ωV/F, variance of inter-individual variability for V/F; ωCL/F, variance of inter-individual variability for CL/F; ωK_a_ variance of inter-individual variability for Ka; ω T_lag_, variance of inter-individual variability for T_lag_; stdev0, standard deviation.

**FIGURE 4:**
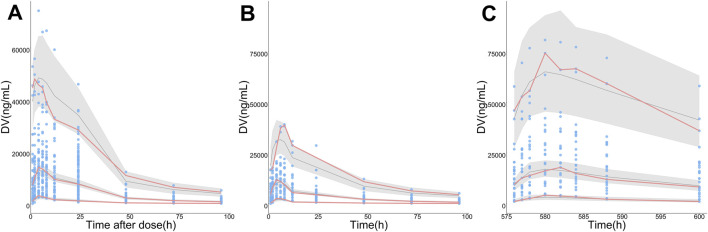
Visual predictive check (VPC) plots for the observations (DV) of ORIN1001. **(A)** DV *versus* time since the nearest administration (Time after dose), **(B)** DV *versus* time after the first administration (0–96 h, pharmacokinetic induction period), **(C)** DV *versus* time after the first administration (576–600 h, the last administration of continuous administration period, day 21 in cycle 1). The sky-blue points represent DV, while the pink solid lines represent the fifth, 50th, and 95th percentiles of DV. The black solid lines show the fifth, 50th and 95th percentiles of simulations, and the shaded regions represent their 95% confidence intervals.

**FIGURE 5:**
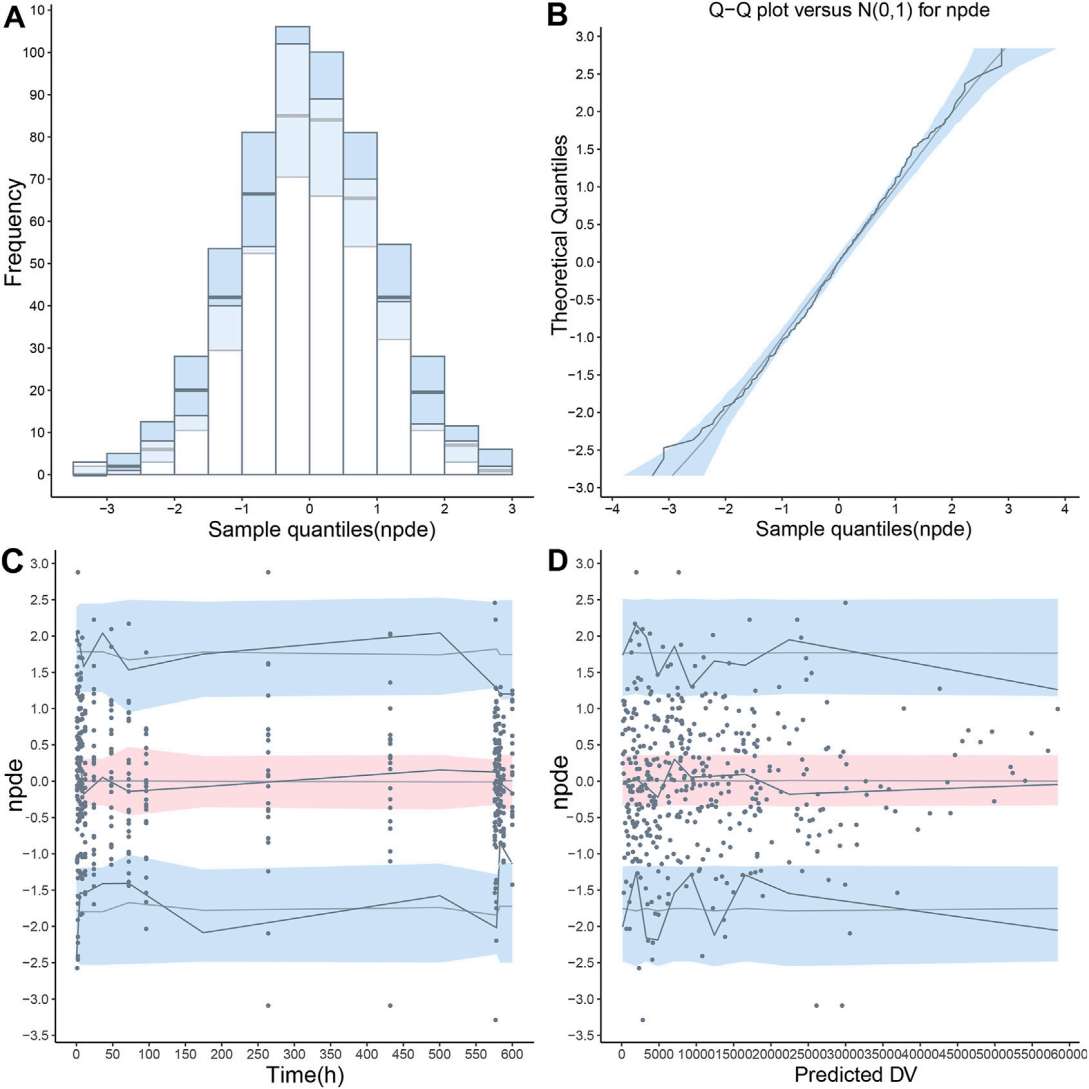
The normalized prediction distribution error (NPDE) value was calculated using the final population pharmacokinetic model. **(A)** Distribution histogram of NPDE value compared with the ideal standard normal distribution (sky blue histogram), **(B)** Quantile-quantile (QQ) plot of NPDE value against theoretical standard normal distribution (sky blue shade), **(C)** A scatterplot presenting NPDE value *versus* time, **(D)** A scatterplot presenting NPDE value *versus* population predictions (PRED). In the last two scatterplots, the observations were shown as blue points. The fifth, 50th and 95th percentiles of observations were shown as solid blue lines, and their 95% confidence intervals were shown as pink or sky-blue fields.

The final PopPK model was also proved to be reliable and robust by the results of other model validation, including GOF plots, VPC, non-parametric bootstrap and NPDE. GOF plots are shown in Supplementary Figure S3. Observations (DV) *versus* PRED or IPRED plots were symmetrically distributed around the solid line (y = x), and CWRES *versus* PRED or time plots showed a random distribution of data points around the horizontal axis (y = 0) between −2 and 2, so no obvious model misspecifications were monitored. VPC plots (Figure 4) showed that the fifth, 50th and 95th quantiles of predicted values lie within the 95% CIs of the corresponding quantiles of the observations, demonstrating the excellent predictive performance of the final model. The VPC plots of the base model are shown in Supplementary Figure S4, indicating that the final model was proved to have a superior capacity than the base model in prediction accuracy. The estimations of final parameters were within the 95% CI of bootstrap predictions (Table 4), indicating the stability of the final PopPK model. NPDE values (Figure 5) followed the standard normal distribution, expressed by their even distribution around 0 and falling within the range of −1.96 to 1.96 (Statistical summary: Student’s t-test, *p* > 0.99; Fisher test, *p* = 0.682; Shapiro-Wilks test of normality, *p* = 0.51), which indicated the good predictability of the final model.

### 3.4 Model simulation

**FIGURE 6:**
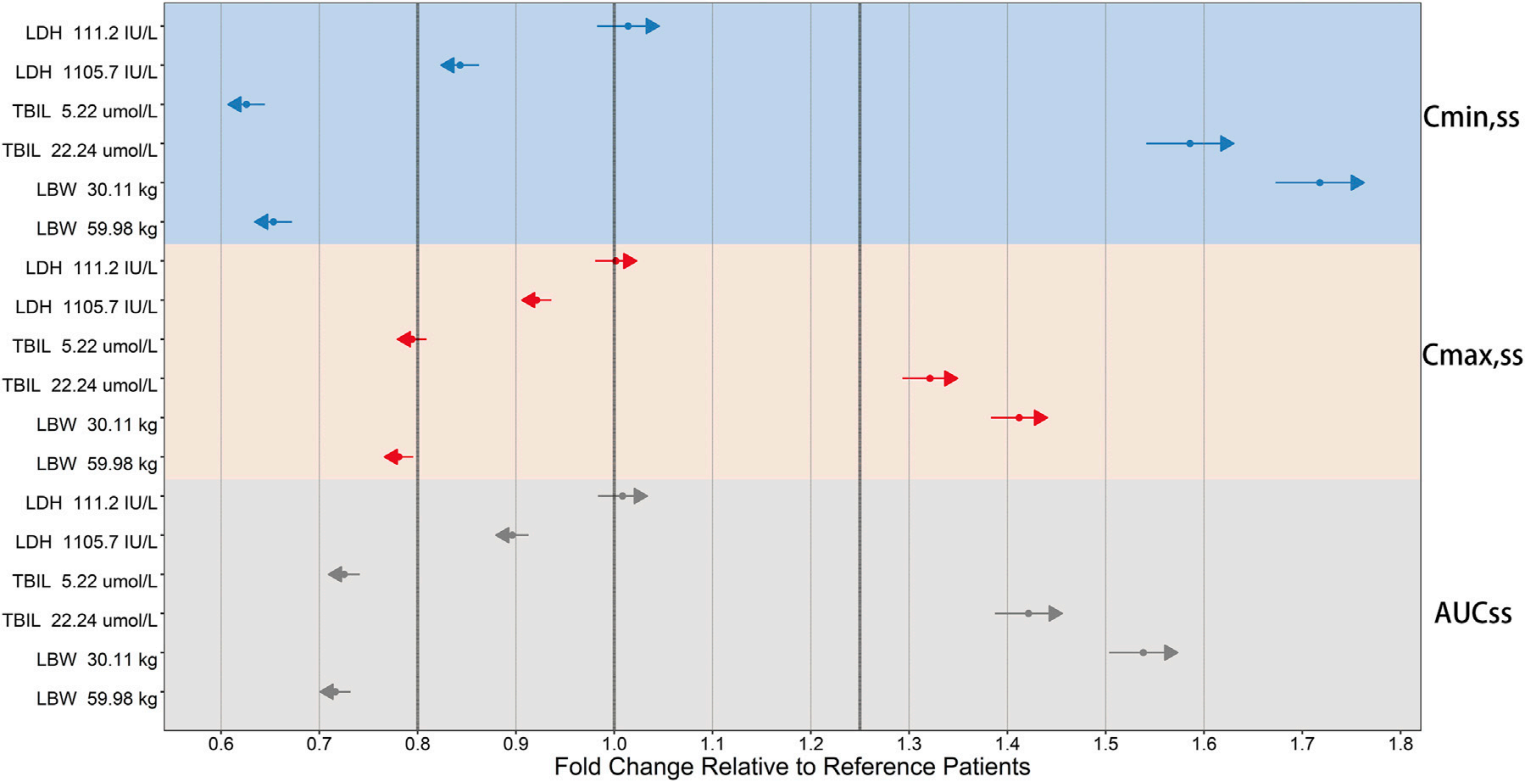
Forest plot of the influence of significant covariates on ORIN1001 steady-state exposure in patients with advanced solid tumors. Steady-state exposure indicators: AUC_ss_, the area under the drug concentration-time profiles; C_max,ss_, the maximum blood drug concentration; C_min,ss_, the minimum blood drug concentration. The sky blue, pink, and gray backgrounds represent the influence of covariates on C_min,ss_, C_max,ss_ and AUC_ss_, respectively. Reference patients: lactate dehydrogenase (LDH) of 214 IU/L, total bilirubin (TBIL) of 10.3 μmol/L and lean body weight (LBW) of 45.13 kg. The covariate values for simulated patients were fixed at the 5th and 95th percentiles of the distribution of the modeling dataset. Calculate their steady-state exposure indicators and obtain the fold changes in the simulated population relative to the reference population. The fold changes of 0.8, 1 and 1.25 are shown as the three thick black dashed lines. The solid points and length of the arrows represent the mean and 95% confidence interval for the fold change (if the mean is greater than 1, the arrows point to the right; otherwise, they point to the left).

The clinically meaningful effect of TBIL and LBW on ORIN1001 steady-state exposure was observed after the simulation (Figure 6). Compared with typical 45.13 kg (median, LBW) patients, C_min,ss_ was 34.7% lower in 59.98 kg patients (95th percentile, LBW) and 71.8% higher in 30.11 kg patients (fifth percentile, LBW). Compared with reference patients with TBIL of 10.3 μmol/L, C_min,ss_ was 37.4% lower in patients with TBIL of 5.22 μmol/L (fifth percentile) and 58.6% higher in patients with TBIL of 22.24 μmol/L (95th percentile). The impact of LBW and TBIL on C_max,ss_ and AUC_ss_ was identified to be similar to that on C_min,ss_, but to a lesser extent.

## 4 Discussion

ORIN1001 is a first-in-class IRE1-α RNase inhibitor to treat tumors by blocking the XBP1 activation selectively and the drug is under clinical research currently. It is necessary to establish the PopPK model in the early stage of the clinical study because knowing about the sources of ORIN1001 PK differences between individuals will be helpful in guiding dose adjustments, improving efficacy and reducing toxicity. In this PopPK study, a two-compartment model with first-order absorption (with T_lag_)/elimination was selected as the optimum structural model, in which T_lag_, V/F and CL/F were estimated as 0.35 h, 26.21 L and 1.07 L/h, respectively. Following stepwise covariate selection, LDH, TBIL and LBW were considered to have statistically significant impacts on PK, but only TBIL and LBW were found to have clinically meaningful effects on ORIN1001 steady-state exposure.

The elimination of ORIN1001 in rats was associated with the liver and kidneys based on the results from preclinical studies, in which ORIN1001 was found to be mainly metabolized by the liver through aldehyde reduction and excreted through feces (64.4%) and urine (12.2%). Therefore, the liver and kidney functions were predicted to be related to ORIN1001 clearance in human. In this PopPK model, TBIL, an indicator of the metabolic and excretory functions of the liver (Tajiri and Shimizu, 2008), was recognized as a significant covariate affecting CL/F and exposure of ORIN1001. This result keeps pace with similar to that from the preclinical study mentioned above. However, CLcr and adj CLcr, indicators of kidney excretion, were not found to have statistically significant effects on the ORIN1001 PK in this study, partly due to the relatively lower contribution of ORIN1001 excretion through the kidney.

It was reported that LBW is a composite indicator derived from weight and BMI (McLeay et al., 2012). And LBW is more appropriate than body weight to contribute to quantifying variations in liver and kidney clearance among individuals because it facilitates a more effective conceptual transition between body composition and clearance, which has been confirmed in previous studies (Han et al., 2007). In the present study, LBW is also found to be a noteworthy covariate, which is consistent with the results from the previous research.

The limited number of subjects in this model seems to be an unneglected drawback, leading to the limit in the extrapolation and application of the model. However, the dense sampling of subjects can compensate for this disadvantage to some extent, fully utilizing the PK information of patients. In this study, TBIL and LBW were observed to play a significant role in explaining the between-subject variability in ORIN1001 PK. However, the necessity of dose adjustments based on these two covariates remains to be validated in a larger population. The reliability and robustness of the model will be further confirmed by means of another dataset from newly recruited subjects in subsequent research. Then, it will be utilized to simulate the concentration-time curves and forecast PK parameters of diverse subjects across various dosing regimens. The results may provide references for the design of clinical studies and accelerate the research process.

## 5 Conclusion

In this study, the first PopPK model of the novel anti-cancer drug ORIN1001 was successfully constructed in Chinese patients with advanced solid tumors. A two-compartment model with first-order absorption (with T_lag_)/elimination was selected as the best structural model. TBIL and LBW were found to have statistically and clinically significant effects on ORIN1001 PK. This PopPK model will provide an important reference for future research on ORIN1001.

## Data Availability

The datasets presented in this article are not readily available because of the confidentiality of innovative anti-tumor drugs. This article contains the original contributions proposed in the study, and further inquiries can be sought from the corresponding author. Requests to access the datasets should be directed to FY, yf7854@163.com.
