# Supplementary material for: Population pharmacokinetic model for oral ORIN1001 in Chinese patients with advanced solid tumors
Source: Front Pharmacol. 2024 Mar 4;15:1322557. doi: 10.3389/fphar.2024.1322557 (PMC10944885; doi:10.3389/fphar.2024.1322557)
Supplement: Supplementary file 1 [file DataSheet1.PDF]

Supplementary Material

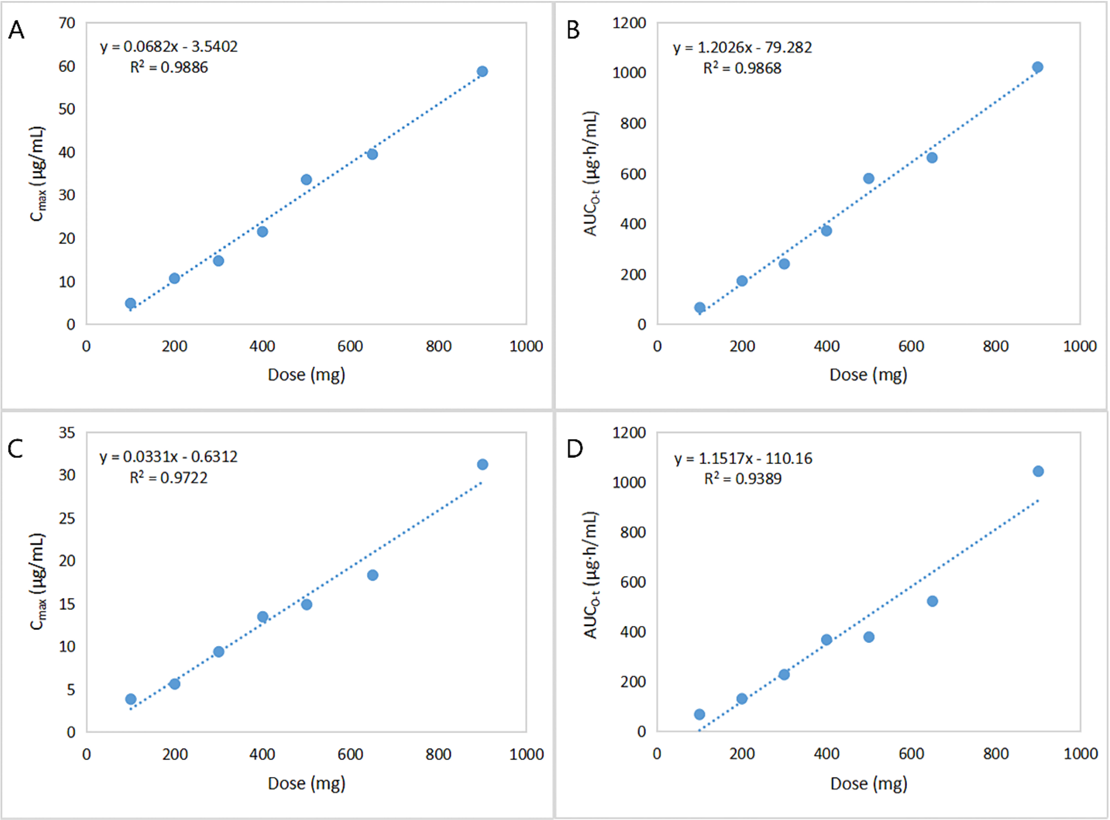

Figure S1 Dose Proportionality of ORIN1001. Figures A (R= 0.99) and B (R= 0.99) are of the multiple-dose, and Figures C (R= 0.99) and D (R= 0.97) are of the single-dose.

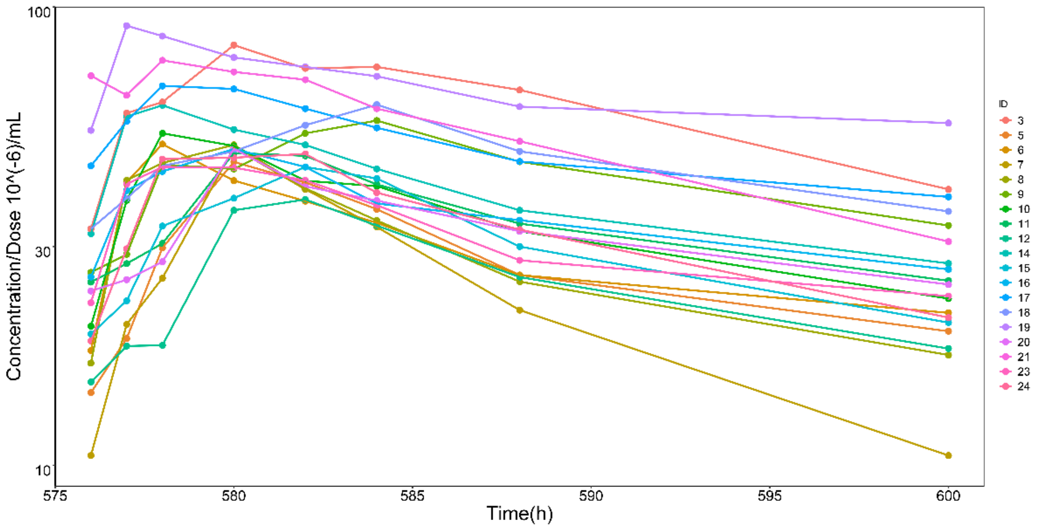

Figure S2 Dose normalized plasma concentration in multiple dosing.

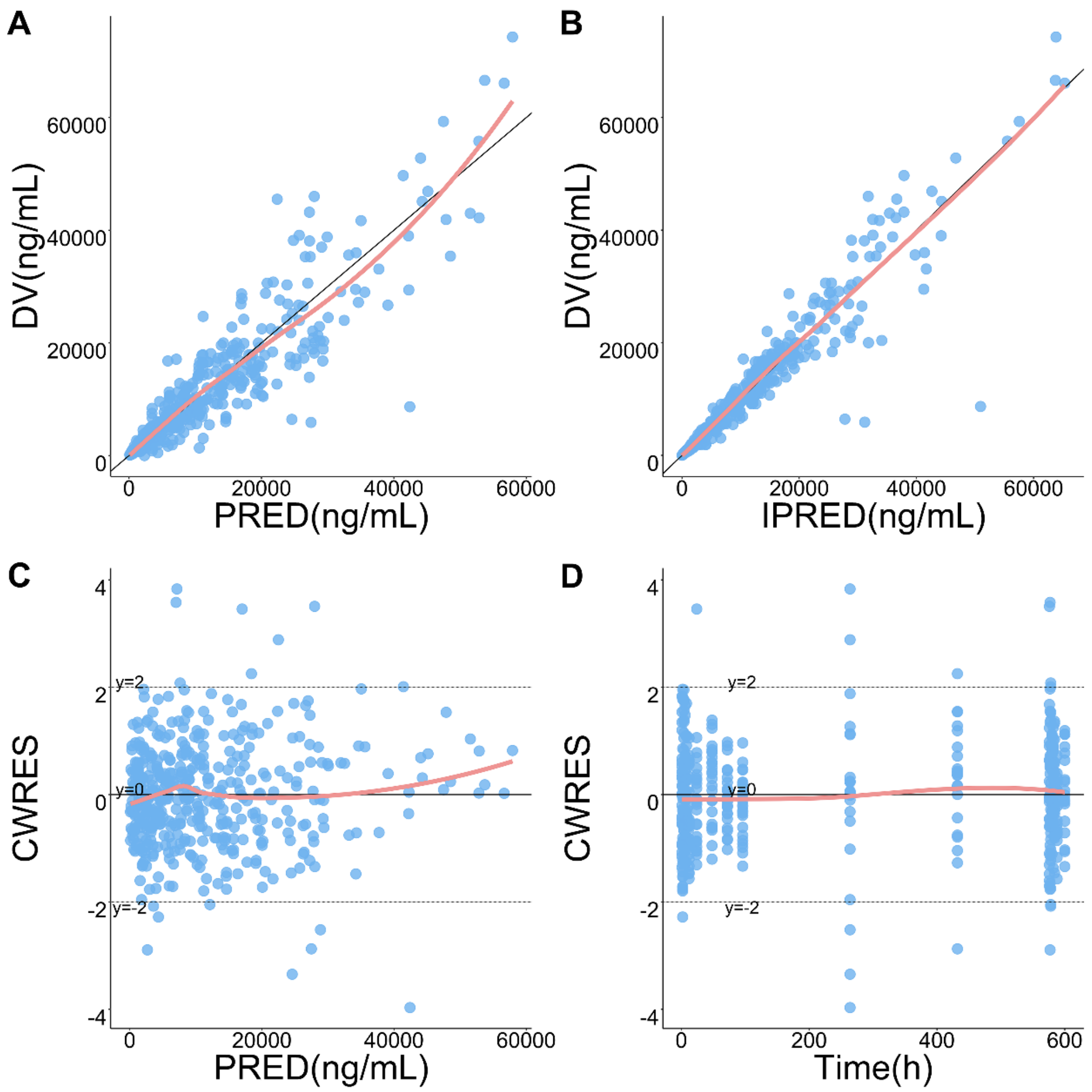

Figure S3 Goodness-of-fit plots of the final population pharmacokinetic model of ORIN1001. (A) Drug concentration observations (DV) versus population predictions (PRED), (B) DV versus individual predictions (IPRED), (C) Conditional weighted residual (CWRES) versus PRED, (D) CWRES value versus time. The pink lines display the loss regression of these points.

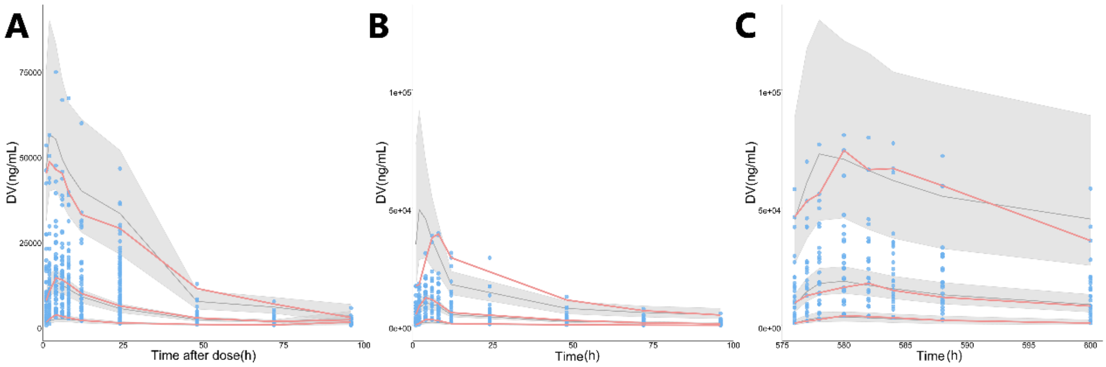

Figure S4 Visual predictive check (VPC) plots of the Base model.

Table S1 The pharmacokinetics parameters in base model

	Base model	
**Parameters**	**Estimate (%RSE)**	**95% CI**
K_a (1/h)	0.68 (12.61%)	0.52-0.85
T_{lag}	0.46 (3.67%)	0.43-0.50
V/F (L)	28.01 (7.10%)	24.10-31.92
V_2/F (L)	25.01 (21.16%)	14.61-35.41
CL/F (L/h)	1.06 (2.83%)	1.00-11.17
CL_2/F (L/h)	0.66 (17.40%)	0.43-0.89
**Inter-individual variability**		
ω^2 V/F	0.030 (31.37%)	0.012-0.048
ω^2 CL/F	0.098 (27.01%)	0.046-0.150
ω^2 K_a	0.628 (23.76%)	0.336-0.921
ω^2 T_{lag}	0.265 (26.80%)	0.126-0.404
**Residual variability (σ)**		
stdev0	0.209 (8.13%)	0.176-0.243

$$CL_2/F\,(L/h) = 0.75 * \left(\frac{LBW}{45.13}\right)^{2.21} \qquad \textit{Eq. S1}$$

$$V/F\,(L) = 26.21 \qquad \textit{Eq. S2}$$

$$V_2/F\,(L) = 26.60 * \left(\frac{LDH}{214}\right)^{0.99} \qquad \textit{Eq. S3}$$

Stepwise Text: key steps have been highlighted in yellow

cstep0000 000000000000000000000000000000000000000000000000, -2LL = 7857.548

Find effect to add that reduces -2LL the most

cstep0001 Ka-BUN 100000000000000000000000000000000000000000000000 7856.918 (7853.077 + 3.841) < 7857.548

cstep0002 Ka-ALP 010000000000000000000000000000000000000000000000 X 7861.389 (7857.547 + 3.841) > 7857.548

cstep0003 Ka-LDH 001000000000000000000000000000000000000000000000 X 7861.391 (7857.550 + 3.841) > 7857.548

cstep0004 Ka-TBIL 000100000000000000000000000000000000000000000000 X 7861.081 (7857.240 + 3.841) > 7857.548

cstep0005 Ka-AST 000010000000000000000000000000000000000000000000 X 7860.844 (7857.003 + 3.841) > 7857.548

cstep0006 Ka-LBW 000001000000000000000000000000000000000000000000 X 7861.354 (7857.513 + 3.841) > 7857.548

cstep0007 Ka-BMI 000000100000000000000000000000000000000000000000 X 7860.193 (7856.352 + 3.841) > 7857.548

cstep0008 Ka-adj CLcr 000000010000000000000000000000000000000000000000 X 7861.386 (7857.545 + 3.841) > 7857.548

cstep0009 V/F-BUN 000000001000000000000000000000000000000000000000 X 7858.923 (7855.082 + 3.841) > 7857.548

cstep0010 V/F-ALP 000000000100000000000000000000000000000000000000 X 7860.702 (7856.861 + 3.841) > 7857.548

cstep0011 V/F-LDH 000000000010000000000000000000000000000000000000 X 7861.378 (7857.537 + 3.841) > 7857.548

cstep0012 V/F-TBIL 000000000001000000000000000000000000000000000000 X 7861.391 (7857.550 + 3.841) > 7857.548

cstep0013 V/F-AST 000000000000100000000000000000000000000000000000 X 7861.061 (7857.220 + 3.841) > 7857.548

cstep0014 V/F-BMI 000000000000010000000000000000000000000000000000 X 7861.391 (7857.550 + 3.841) > 7857.548

cstep0015 V/F-LBW 000000000000001000000000000000000000000000000000 X 7861.182 (7857.341 + 3.841) > 7857.548

cstep0016 V/F-adj CLcr 000000000000000100000000000000000000000000000000 X 7859.818 (7855.977 + 3.841) > 7857.548

cstep0017 V2/F-BUN 000000000000000010000000000000000000000000000000 X 7861.168 (7857.327 + 3.841) > 7857.548

cstep0018 V2/F-ALP 000000000000000001000000000000000000000000000000 X 7860.045 (7856.204 + 3.841) > 7857.548

cstep0019 V2/F-LDH 000000000000000000100000000000000000000000000000 X 7861.019 (7857.178 + 3.841) > 7857.548

cstep0020 V2/F-TBIL 000000000000000000010000000000000000000000000000 X 7861.390 (7857.549 + 3.841) > 7857.548

cstep0021 V2/F-AST 000000000000000000001000000000000000000000000000 X 7861.384 (7857.543 + 3.841) > 7857.548

cstep0022 V2/F-BMI 000000000000000000000100000000000000000000000000 X 7861.384 (7857.543 + 3.841) > 7857.548

cstep0023 V2/F-LBW 000000000000000000000010000000000000000000000000 X 7860.569 (7856.728 + 3.841) > 7857.548

cstep0024 V2/F-adj CLcr 000000000000000000000001000000000000000000000000 X 7861.391 (7857.550 + 3.841) > 7857.548

cstep0025 Cl/F-BUN 000000000000000000000000100000000000000000000000 X 7860.025 (7856.184 + 3.841) > 7857.548

cstep0026 Cl/F-ALP 000000000000000000000000010000000000000000000000 X 7859.962 (7856.120 + 3.841) > 7857.548

cstep0027 Cl/F-LDH 000000000000000000000000001000000000000000000000 X 7861.393 (7857.551 + 3.841) > 7857.548

cstep0028 Cl/F-TBIL 000000000000000000000000000100000000000000000000 7856.067 (7852.225 + 3.841) < 7857.548

cstep0029 Cl/F-AST 000000000000000000000000000010000000000000000000 X 7861.209 (7857.368 + 3.841) > 7857.548

cstep0030 Cl/F-BMI 000000000000000000000000000001000000000000000000 X 7860.575 (7856.734 + 3.841) > 7857.548

cstep0031 Cl/F-LBW 000000000000000000000000000000100000000000000000 X 7858.005 (7854.163 + 3.841) > 7857.548

cstep0032 Cl/F-adj CLcr 000000000000000000000000000000010000000000000000 X 7860.612 (7856.771 + 3.841) > 7857.548

cstep0033 Cl2/F-BUN 000000000000000000000000000000001000000000000000 7849.641 (7845.800 + 3.841) < 7857.548

cstep0034 Cl2/F-ALP 000000000000000000000000000000000100000000000000 X 7860.473 (7856.631 + 3.841) > 7857.548

cstep0035 Cl2/F-LDH 000000000000000000000000000000000010000000000000 X 7859.882 (7856.040 + 3.841) > 7857.548

cstep0036 Cl2/F-TBIL 000000000000000000000000000000000001000000000000 X 7860.754 (7856.913 + 3.841) > 7857.548

cstep0037 Cl2/F-AST 000000000000000000000000000000000000100000000000 X 7861.259 (7857.418 + 3.841) > 7857.548

cstep0038 Cl2/F-BMI 000000000000000000000000000000000000010000000000 X 7861.391 (7857.549 + 3.841) > 7857.548

cstep0039 Cl2/F-LBW 000000000000000000000000000000000000001000000000 7852.741 (7848.900 + 3.841) < 7857.548

cstep0040 Cl2/F-adj CLcr 000000000000000000000000000000000000000100000000 X 7861.199 (7857.357 + 3.841) > 7857.548

cstep0041 Tlag-BUN 000000000000000000000000000000000000000010000000 X 7861.389 (7857.547 + 3.841) > 7857.548

cstep0042 Tlag-ALP 000000000000000000000000000000000000000001000000 X 7861.392 (7857.550 + 3.841) > 7857.548

cstep0043 Tlag-LDH 000000000000000000000000000000000000000000100000 X 7861.199 (7857.358 + 3.841) > 7857.548

cstep0044 Tlag-TBIL 000000000000000000000000000000000000000000010000 X 7858.399 (7854.558 + 3.841) > 7857.548

cstep0045 Tlag-AST 000000000000000000000000000000000000000000001000 X 7861.391 (7857.550 + 3.841) > 7857.548

cstep0046 Tlag-BMI 000000000000000000000000000000000000000000000100 X 7861.388 (7857.547 + 3.841) > 7857.548

cstep0047 Tlag-LBW 000000000000000000000000000000000000000000000010 X 7861.390 (7857.549 + 3.841) > 7857.548

cstep0048 Tlag-adj CLcr 000000000000000000000000000000000000000000000001 X 7860.859 (7857.018 + 3.841) > 7857.548

cstep0033 Cl2/F-BUN 000000000000000000000000000000001000000000000000 chosen, -2LL = 7845.800

Find effect to add that reduces -2LL the most

cstep0049 Ka-BUN Cl2/F-BUN 100000000000000000000000000000001000000000000000 X 7846.303
(7842.462 + 3.841) > 7845.800
cstep0050 Ka-ALP Cl2/F-BUN 010000000000000000000000000000001000000000000000 X 7849.603
(7845.761 + 3.841) > 7845.800
cstep0051 Ka-LDH Cl2/F-BUN 001000000000000000000000000000001000000000000000 X 7849.477
(7845.636 + 3.841) > 7845.800
cstep0052 Ka-TBIL Cl2/F-BUN 000100000000000000000000000000001000000000000000 X 7849.271
(7845.430 + 3.841) > 7845.800
cstep0053 Ka-AST Cl2/F-BUN 000010000000000000000000000000001000000000000000 X 7848.834
(7844.992 + 3.841) > 7845.800
cstep0054 Ka-LBW Cl2/F-BUN 000001000000000000000000000000001000000000000000 X 7848.366
(7844.525 + 3.841) > 7845.800
cstep0055 Ka-BMI Cl2/F-BUN 000000100000000000000000000000001000000000000000 X 7848.365
(7844.523 + 3.841) > 7845.800
cstep0056 Ka-adj CLcr Cl2/F-BUN 000000010000000000000000000000001000000000000000 X 7850.489
(7846.647 + 3.841) > 7845.800
cstep0057 V/F-BUN Cl2/F-BUN 000000001000000000000000000000001000000000000000 X 7849.550
(7845.709 + 3.841) > 7845.800
cstep0058 V/F-ALP Cl2/F-BUN 000000000100000000000000000000001000000000000000 X 7849.545
(7845.704 + 3.841) > 7845.800
cstep0059 V/F-LDH Cl2/F-BUN 000000000010000000000000000000001000000000000000 X 7849.181
(7845.339 + 3.841) > 7845.800
cstep0060 V/F-TBIL Cl2/F-BUN 000000000001000000000000000000001000000000000000 X 7849.293
(7845.451 + 3.841) > 7845.800
cstep0061 V/F-AST Cl2/F-BUN 000000000000100000000000000000001000000000000000 X 7849.628
(7845.787 + 3.841) > 7845.800
cstep0062 V/F-BMI Cl2/F-BUN 000000000000010000000000000000001000000000000000 X 7848.267
(7844.425 + 3.841) > 7845.800
cstep0063 V/F-LBW Cl2/F-BUN 000000000000001000000000000000001000000000000000 X 7849.617
(7845.776 + 3.841) > 7845.800
cstep0064 V/F-adj CLcr Cl2/F-BUN 000000000000000100000000000000001000000000000000 X 7848.757
(7844.916 + 3.841) > 7845.800
cstep0065 V2/F-BUN Cl2/F-BUN 000000000000000010000000000000001000000000000000 X 7849.417
(7845.576 + 3.841) > 7845.800
cstep0066 V2/F-ALP Cl2/F-BUN 000000000000000001000000000000001000000000000000 X 7847.355
(7843.513 + 3.841) > 7845.800
cstep0067 V2/F-LDH Cl2/F-BUN 000000000000000000100000000000001000000000000000 X 7845.974
(7842.132 + 3.841) > 7845.800
cstep0068 V2/F-TBIL Cl2/F-BUN 000000000000000000010000000000001000000000000000 X 7849.692
(7845.851 + 3.841) > 7845.800
cstep0069 V2/F-AST Cl2/F-BUN 000000000000000000001000000000001000000000000000 X 7848.250
(7844.409 + 3.841) > 7845.800
cstep0070 V2/F-BMI Cl2/F-BUN 000000000000000000000100000000001000000000000000 X 7849.390
(7845.549 + 3.841) > 7845.800

cstep0071 V2/F-LBW Cl2/F-BUN 000000000000000000000010000000001000000000000000 X 7849.065

(7845.223 + 3.841) > 7845.800

cstep0072 V2/F-adj CLcr Cl2/F-BUN 000000000000000000000001000000001000000000000000 X 7849.704

(7845.863 + 3.841) > 7845.800

cstep0073 Cl/F-BUN Cl2/F-BUN 000000000000000000000000100000001000000000000000 X 7848.109

(7844.267 + 3.841) > 7845.800

cstep0074 Cl/F-ALP Cl2/F-BUN 000000000000000000000000010000001000000000000000 X 7847.863

(7844.021 + 3.841) > 7845.800

cstep0075 Cl/F-LDH Cl2/F-BUN 000000000000000000000000001000001000000000000000 X 7849.497

(7845.655 + 3.841) > 7845.800

cstep0076 Cl/F-TBIL Cl2/F-BUN 000000000000000000000000000100001000000000000000 7844.352

(7840.511 + 3.841) < 7845.800

cstep0077 Cl/F-AST Cl2/F-BUN 000000000000000000000000000010001000000000000000 X 7848.999

(7845.157 + 3.841) > 7845.800

cstep0078 Cl/F-BMI Cl2/F-BUN 000000000000000000000000000001001000000000000000 X 7848.765

(7844.923 + 3.841) > 7845.800

cstep0079 Cl/F-LBW Cl2/F-BUN 000000000000000000000000000000101000000000000000 X 7845.914

(7842.073 + 3.841) > 7845.800

cstep0080 Cl/F-adj CLcr Cl2/F-BUN 000000000000000000000000000000011000000000000000 X 7848.577

(7844.735 + 3.841) > 7845.800

cstep0081 Cl2/F-BUN Cl2/F-ALP 000000000000000000000000000000001100000000000000 X 7848.464

(7844.622 + 3.841) > 7845.800

cstep0082 Cl2/F-BUN Cl2/F-LDH 000000000000000000000000000000001010000000000000 7845.748

(7841.907 + 3.841) < 7845.800

cstep0083 Cl2/F-BUN Cl2/F-TBIL 000000000000000000000000000000001001000000000000 X 7849.174

(7845.333 + 3.841) > 7845.800

cstep0084 Cl2/F-BUN Cl2/F-AST 000000000000000000000000000000001000100000000000 X 7849.576

(7845.735 + 3.841) > 7845.800

cstep0085 Cl2/F-BUN Cl2/F-BMI 000000000000000000000000000000001000010000000000 X 7849.220

(7845.378 + 3.841) > 7845.800

cstep0086 Cl2/F-BUN Cl2/F-LBW 000000000000000000000000000000001000001000000000 X 7849.186

(7845.345 + 3.841) > 7845.800

cstep0087 Cl2/F-BUN Cl2/F-adj CLcr 000000000000000000000000000000001000000100000000 X 7849.547

(7845.705 + 3.841) > 7845.800

cstep0088 Cl2/F-BUN Tlag-BUN 000000000000000000000000000000001000000010000000 X 7848.668

(7844.827 + 3.841) > 7845.800

cstep0089 Cl2/F-BUN Tlag-ALP 000000000000000000000000000000001000000001000000 X 7849.626

(7845.785 + 3.841) > 7845.800

cstep0090 Cl2/F-BUN Tlag-LDH 000000000000000000000000000000001000000000100000 X 7849.480

(7845.638 + 3.841) > 7845.800

cstep0091 Cl2/F-BUN Tlag-TBIL 000000000000000000000000000000001000000000010000 X 7846.512

(7842.670 + 3.841) > 7845.800

cstep0092 Cl2/F-BUN Tlag-AST 000000000000000000000000000000001000000000001000 X 7850.448

(7846.607 + 3.841) > 7845.800

cstep0093 Cl2/F-BUN Tlag-BMI 000000000000000000000000000000000001000000000000100 X 7846.685 (7842.844 + 3.841) > 7845.800

cstep0094 Cl2/F-BUN Tlag-LBW 000000000000000000000000000000000001000000000000010 X 7849.548 (7845.706 + 3.841) > 7845.800

cstep0095 Cl2/F-BUN Tlag-adj CLcr 000000000000000000000000000000000001000000000000001 X 7849.087 (7845.246 + 3.841) > 7845.800

cstep0076 Cl/F-TBIL Cl2/F-BUN 000000000000000000000000000000100001000000000000000 chosen, -2LL = 7840.511

Find effect to add that reduces -2LL the most

cstep0096 Ka-BUN Cl/F-TBIL Cl2/F-BUN 100000000000000000000000000000100001000000000000000 X 7840.975 (7837.134 + 3.841) > 7840.511

cstep0097 Ka-ALP Cl/F-TBIL Cl2/F-BUN 010000000000000000000000000000100001000000000000000 X 7844.312 (7840.470 + 3.841) > 7840.511

cstep0098 Ka-LDH Cl/F-TBIL Cl2/F-BUN 001000000000000000000000000000100001000000000000000 X 7844.282 (7840.440 + 3.841) > 7840.511

cstep0099 Ka-TBIL Cl/F-TBIL Cl2/F-BUN 000100000000000000000000000000100001000000000000000 X 7844.008 (7840.167 + 3.841) > 7840.511

cstep0100 Ka-AST Cl/F-TBIL Cl2/F-BUN 000010000000000000000000000000100001000000000000000 X 7843.581 (7839.740 + 3.841) > 7840.511

cstep0101 Ka-LBW Cl/F-TBIL Cl2/F-BUN 000001000000000000000000000000100001000000000000000 X 7843.115 (7839.273 + 3.841) > 7840.511

cstep0102 Ka-BMI Cl/F-TBIL Cl2/F-BUN 000000100000000000000000000000100001000000000000000 X 7843.828 (7839.987 + 3.841) > 7840.511

cstep0103 Ka-adj CLcr Cl/F-TBIL Cl2/F-BUN 000000010000000000000000000000100001000000000000000 X 7844.073 (7840.231 + 3.841) > 7840.511

cstep0104 V/F-BUN Cl/F-TBIL Cl2/F-BUN 000000001000000000000000000000100001000000000000000 X 7844.246 (7840.405 + 3.841) > 7840.511

cstep0105 V/F-ALP Cl/F-TBIL Cl2/F-BUN 000000000100000000000000000000100001000000000000000 X 7844.284 (7840.442 + 3.841) > 7840.511

cstep0106 V/F-LDH Cl/F-TBIL Cl2/F-BUN 000000000010000000000000000000100001000000000000000 X 7843.927 (7840.086 + 3.841) > 7840.511

cstep0107 V/F-TBIL Cl/F-TBIL Cl2/F-BUN 000000000001000000000000000000100001000000000000000 X 7844.458 (7840.617 + 3.841) > 7840.511

cstep0108 V/F-AST Cl/F-TBIL Cl2/F-BUN 000000000000100000000000000000100001000000000000000 X 7844.337 (7840.495 + 3.841) > 7840.511

cstep0109 V/F-BMI Cl/F-TBIL Cl2/F-BUN 000000000000010000000000000000100001000000000000000 X 7843.040 (7839.198 + 3.841) > 7840.511

cstep0110 V/F-LBW Cl/F-TBIL Cl2/F-BUN 000000000000001000000000000000100001000000000000000 X 7844.723 (7840.881 + 3.841) > 7840.511

cstep0111 V/F-adj CLcr Cl/F-TBIL Cl2/F-BUN 000000000000000100000000000000100001000000000000000 X 7843.431 (7839.590 + 3.841) > 7840.511

cstep0112 V2/F-BUN Cl/F-TBIL Cl2/F-BUN 000000000000000010000000000000100001000000000000000 X

7843.983 (7840.141 + 3.841) > 7840.511

cstep0113 V2/F-ALP Cl/F-TBIL Cl2/F-BUN 0000000000000000001000000000100001000000000000000 X

7842.073 (7838.232 + 3.841) > 7840.511

cstep0114 V2/F-LDH Cl/F-TBIL Cl2/F-BUN 0000000000000000000100000000100001000000000000000

7839.602 (7835.761 + 3.841) < 7840.511

cstep0115 V2/F-TBIL Cl/F-TBIL Cl2/F-BUN 0000000000000000000010000000100001000000000000000 X

7844.428 (7840.587 + 3.841) > 7840.511

cstep0116 V2/F-AST Cl/F-TBIL Cl2/F-BUN 0000000000000000000001000000100001000000000000000 X

7842.415 (7838.574 + 3.841) > 7840.511

cstep0117 V2/F-BMI Cl/F-TBIL Cl2/F-BUN 0000000000000000000000100000100001000000000000000 X

7843.969 (7840.128 + 3.841) > 7840.511

cstep0118 V2/F-LBW Cl/F-TBIL Cl2/F-BUN 0000000000000000000000010000100001000000000000000 X

7843.695 (7839.854 + 3.841) > 7840.511

cstep0119 V2/F-adj CLcr Cl/F-TBIL Cl2/F-BUN 0000000000000000000000001000100001000000000000000 X 7844.771 (7840.929 + 3.841) > 7840.511

cstep0120 Cl/F-BUN Cl/F-TBIL Cl2/F-BUN 0000000000000000000000000100100001000000000000000 X

7842.711 (7838.870 + 3.841) > 7840.511

cstep0121 Cl/F-ALP Cl/F-TBIL Cl2/F-BUN 0000000000000000000000000010100001000000000000000 X

7843.328 (7839.487 + 3.841) > 7840.511

cstep0122 Cl/F-LDH Cl/F-TBIL Cl2/F-BUN 0000000000000000000000000001100001000000000000000 X

7844.807 (7840.966 + 3.841) > 7840.511

cstep0123 Cl/F-TBIL Cl/F-AST Cl2/F-BUN 0000000000000000000000000000110001000000000000000 X

7844.372 (7840.530 + 3.841) > 7840.511

cstep0124 Cl/F-TBIL Cl/F-BMI Cl2/F-BUN 0000000000000000000000000000101001000000000000000 X

7841.965 (7838.123 + 3.841) > 7840.511

cstep0125 Cl/F-TBIL Cl/F-LBW Cl2/F-BUN 0000000000000000000000000000100101000000000000000

7838.963 (7835.122 + 3.841) < 7840.511

cstep0126 Cl/F-TBIL Cl/F-adj CLcr Cl2/F-BUN 0000000000000000000000000000100011000000000000000 X

7842.977 (7839.135 + 3.841) > 7840.511

cstep0127 Cl/F-TBIL Cl2/F-BUN Cl2/F-ALP 0000000000000000000000000000100001100000000000000 X

7847.787 (7843.946 + 3.841) > 7840.511

cstep0128 Cl/F-TBIL Cl2/F-BUN Cl2/F-LDH 0000000000000000000000000000100001010000000000000 X

7840.620 (7836.779 + 3.841) > 7840.511

cstep0129 Cl/F-TBIL Cl2/F-BUN Cl2/F-TBIL 0000000000000000000000000000100001001000000000000 X

7843.709 (7839.867 + 3.841) > 7840.511

cstep0130 Cl/F-TBIL Cl2/F-BUN Cl2/F-AST 0000000000000000000000000000100001000100000000000 X

7844.259 (7840.417 + 3.841) > 7840.511

cstep0131 Cl/F-TBIL Cl2/F-BUN Cl2/F-BMI 0000000000000000000000000000100001000010000000000 X

7844.135 (7840.294 + 3.841) > 7840.511

cstep0132 Cl/F-TBIL Cl2/F-BUN Cl2/F-LBW 0000000000000000000000000000100001000001000000000 X

7843.950 (7840.109 + 3.841) > 7840.511

cstep0133 Cl/F-TBIL Cl2/F-BUN Cl2/F-adj CLcr 0000000000000000000000000000100001000000100000000 X 7844.318 (7840.477 + 3.841) > 7840.511

cstep0134 Cl/F-TBIL Cl2/F-BUN Tlag-BUN 0000000000000000000000000000100001000000010000000 X

7843.524 (7839.682 + 3.841) > 7840.511

cstep0135 Cl/F-TBIL Cl2/F-BUN Tlag-ALP 000000000000000000000000000100001000000001000000 X 7844.320 (7840.478 + 3.841) > 7840.511

cstep0136 Cl/F-TBIL Cl2/F-BUN Tlag-LDH 000000000000000000000000000100001000000000100000 X 7844.204 (7840.363 + 3.841) > 7840.511

cstep0137 Cl/F-TBIL Cl2/F-BUN Tlag-TBIL 000000000000000000000000000100001000000000010000 X 7841.127 (7837.285 + 3.841) > 7840.511

cstep0138 Cl/F-TBIL Cl2/F-BUN Tlag-AST 000000000000000000000000000100001000000000001000 X 7842.982 (7839.140 + 3.841) > 7840.511

cstep0139 Cl/F-TBIL Cl2/F-BUN Tlag-BMI 000000000000000000000000000100001000000000000100 X 7840.728 (7836.886 + 3.841) > 7840.511

cstep0140 Cl/F-TBIL Cl2/F-BUN Tlag-LBW 000000000000000000000000000100001000000000000010 X 7844.403 (7840.562 + 3.841) > 7840.511

cstep0141 Cl/F-TBIL Cl2/F-BUN Tlag-adj CLcr 000000000000000000000000000100001000000000000001 X 7843.788 (7839.947 + 3.841) > 7840.511

cstep0125 Cl/F-TBIL Cl/F-LBW Cl2/F-BUN 000000000000000000000000000100101000000000000000 chosen, -2LL = 7835.122

Find effect to add that reduces -2LL the most

cstep0142 Ka-BUN Cl/F-TBIL Cl/F-LBW Cl2/F-BUN 100000000000000000000000000100101000000000000000 X 7835.470 (7831.628 + 3.841) > 7835.122

cstep0143 Ka-ALP Cl/F-TBIL Cl/F-LBW Cl2/F-BUN 010000000000000000000000000100101000000000000000 X 7838.903 (7835.061 + 3.841) > 7835.122

cstep0144 Ka-LDH Cl/F-TBIL Cl/F-LBW Cl2/F-BUN 001000000000000000000000000100101000000000000000 X 7840.213 (7836.372 + 3.841) > 7835.122

cstep0145 Ka-TBIL Cl/F-TBIL Cl/F-LBW Cl2/F-BUN 000100000000000000000000000100101000000000000000 X 7838.608 (7834.766 + 3.841) > 7835.122

cstep0146 Ka-AST Cl/F-TBIL Cl/F-LBW Cl2/F-BUN 000010000000000000000000000100101000000000000000 X 7838.661 (7834.820 + 3.841) > 7835.122

cstep0147 Ka-LBW Cl/F-TBIL Cl/F-LBW Cl2/F-BUN 000001000000000000000000000100101000000000000000 X 7837.698 (7833.856 + 3.841) > 7835.122

cstep0148 Ka-BMI Cl/F-TBIL Cl/F-LBW Cl2/F-BUN 000000100000000000000000000100101000000000000000 X 7838.063 (7834.221 + 3.841) > 7835.122

cstep0149 Ka-adj CLcr Cl/F-TBIL Cl/F-LBW Cl2/F-BUN 000000010000000000000000000100101000000000000000 X 7838.621 (7834.779 + 3.841) > 7835.122

cstep0150 V/F-BUN Cl/F-TBIL Cl/F-LBW Cl2/F-BUN 000000001000000000000000000100101000000000000000 X 7838.593 (7834.751 + 3.841) > 7835.122

cstep0151 V/F-ALP Cl/F-TBIL Cl/F-LBW Cl2/F-BUN 000000000100000000000000000100101000000000000000 X 7838.795 (7834.954 + 3.841) > 7835.122

cstep0152 V/F-LDH Cl/F-TBIL Cl/F-LBW Cl2/F-BUN 000000000010000000000000000100101000000000000000 X 7838.439 (7834.598 + 3.841) > 7835.122

cstep0153 V/F-TBIL Cl/F-TBIL Cl/F-LBW Cl2/F-BUN 000000000001000000000000000100101000000000000000 X 7838.651 (7834.809 + 3.841) > 7835.122

cstep0154 V/F-AST Cl/F-TBIL Cl/F-LBW Cl2/F-BUN 0000000000001000000000000010010100000000000000000 X 7839.034 (7835.193 + 3.841) > 7835.122

cstep0155 V/F-BMI Cl/F-TBIL Cl/F-LBW Cl2/F-BUN 0000000000000100000000000010010100000000000000000 X 7837.622 (7833.781 + 3.841) > 7835.122

cstep0156 V/F-LBW Cl/F-TBIL Cl/F-LBW Cl2/F-BUN 00000000000001000000000000100101000000000000000 X 7839.245 (7835.404 + 3.841) > 7835.122

cstep0157 V/F-adj CLcr Cl/F-TBIL Cl/F-LBW Cl2/F-BUN 00000000000000100000000000100101000000000000000 X 7838.007 (7834.165 + 3.841) > 7835.122

cstep0158 V2/F-BUN Cl/F-TBIL Cl/F-LBW Cl2/F-BUN 00000000000000010000000000100101000000000000000 X 7838.865 (7835.024 + 3.841) > 7835.122

cstep0159 V2/F-ALP Cl/F-TBIL Cl/F-LBW Cl2/F-BUN 00000000000000001000000000100101000000000000000 X 7836.389 (7832.548 + 3.841) > 7835.122

cstep0160 V2/F-LDH Cl/F-TBIL Cl/F-LBW Cl2/F-BUN 00000000000000000100000000100101000000000000000 7833.019 (7829.178 + 3.841) < 7835.122

cstep0161 V2/F-TBIL Cl/F-TBIL Cl/F-LBW Cl2/F-BUN 00000000000000000010000000100101000000000000000 X 7838.822 (7834.981 + 3.841) > 7835.122

cstep0162 V2/F-AST Cl/F-TBIL Cl/F-LBW Cl2/F-BUN 00000000000000000001000000100101000000000000000 X 7836.116 (7832.275 + 3.841) > 7835.122

cstep0163 V2/F-BMI Cl/F-TBIL Cl/F-LBW Cl2/F-BUN 00000000000000000000100000100101000000000000000 X 7838.506 (7834.664 + 3.841) > 7835.122

cstep0164 V2/F-LBW Cl/F-TBIL Cl/F-LBW Cl2/F-BUN 00000000000000000000010000100101000000000000000 X 7838.818 (7834.977 + 3.841) > 7835.122

cstep0165 V2/F-adj CLcr Cl/F-TBIL Cl/F-LBW Cl2/F-BUN 00000000000000000000001000100101000000000000000 X 7838.945 (7835.104 + 3.841) > 7835.122

cstep0166 Cl/F-BUN Cl/F-TBIL Cl/F-LBW Cl2/F-BUN 00000000000000000000000100100101000000000000000 X 7839.243 (7835.401 + 3.841) > 7835.122

cstep0167 Cl/F-ALP Cl/F-TBIL Cl/F-LBW Cl2/F-BUN 00000000000000000000000010100101000000000000000 X 7838.058 (7834.217 + 3.841) > 7835.122

cstep0168 Cl/F-LDH Cl/F-TBIL Cl/F-LBW Cl2/F-BUN 00000000000000000000000001100101000000000000000 X 7838.889 (7835.047 + 3.841) > 7835.122

cstep0169 Cl/F-TBIL Cl/F-AST Cl/F-LBW Cl2/F-BUN 00000000000000000000000000110101000000000000000 X 7838.983 (7835.142 + 3.841) > 7835.122

cstep0170 Cl/F-TBIL Cl/F-BMI Cl/F-LBW Cl2/F-BUN 00000000000000000000000000101101000000000000000 X 7836.677 (7832.836 + 3.841) > 7835.122

cstep0171 Cl/F-TBIL Cl/F-LBW Cl/F-adj CLcr Cl2/F-BUN 00000000000000000000000000100111000000000000000 X 7838.802 (7834.960 + 3.841) > 7835.122

cstep0172 Cl/F-TBIL Cl/F-LBW Cl2/F-BUN Cl2/F-ALP 00000000000000000000000000100101100000000000000 X 7837.837 (7833.996 + 3.841) > 7835.122

cstep0173 Cl/F-TBIL Cl/F-LBW Cl2/F-BUN Cl2/F-LDH 00000000000000000000000000100101010000000000000 X 7836.365 (7832.524 + 3.841) > 7835.122

cstep0174 Cl/F-TBIL Cl/F-LBW Cl2/F-BUN Cl2/F-TBIL 00000000000000000000000000100101001000000000000 X 7838.447 (7834.605 + 3.841) > 7835.122

cstep0175 Cl/F-TBIL Cl/F-LBW Cl2/F-BUN Cl2/F-AST 00000000000000000000000000100101000100000000000 X 7838.823 (7834.981 + 3.841) > 7835.122

cstep0176 Cl/F-TBIL Cl/F-LBW Cl2/F-BUN Cl2/F-BMI

000000000000000000000000000100101000010000000000 X 7838.523 (7834.681 + 3.841) > 7835.122

cstep0177 Cl/F-TBIL Cl/F-LBW Cl2/F-BUN Cl2/F-LBW

000000000000000000000000000100101000001000000000 X 7838.303 (7834.461 + 3.841) > 7835.122

cstep0178 Cl/F-TBIL Cl/F-LBW Cl2/F-BUN Cl2/F-adj CLcr

000000000000000000000000000100101000000100000000 X 7838.825 (7834.983 + 3.841) > 7835.122

cstep0179 Cl/F-TBIL Cl/F-LBW Cl2/F-BUN Tlag-BUN

000000000000000000000000000100101000000010000000 X 7837.972 (7834.131 + 3.841) > 7835.122

cstep0180 Cl/F-TBIL Cl/F-LBW Cl2/F-BUN Tlag-ALP

000000000000000000000000000100101000000001000000 X 7838.912 (7835.070 + 3.841) > 7835.122

cstep0181 Cl/F-TBIL Cl/F-LBW Cl2/F-BUN Tlag-LDH

000000000000000000000000000100101000000000100000 X 7838.698 (7834.857 + 3.841) > 7835.122

cstep0182 Cl/F-TBIL Cl/F-LBW Cl2/F-BUN Tlag-TBIL

000000000000000000000000000100101000000000010000 X 7835.555 (7831.713 + 3.841) > 7835.122

cstep0183 Cl/F-TBIL Cl/F-LBW Cl2/F-BUN Tlag-AST

000000000000000000000000000100101000000000001000 X 7837.501 (7833.659 + 3.841) > 7835.122

cstep0184 Cl/F-TBIL Cl/F-LBW Cl2/F-BUN Tlag-BMI

000000000000000000000000000100101000000000000100 X 7835.199 (7831.357 + 3.841) > 7835.122

cstep0185 Cl/F-TBIL Cl/F-LBW Cl2/F-BUN Tlag-LBW

000000000000000000000000000100101000000000000010 X 7839.118 (7835.277 + 3.841) > 7835.122

cstep0186 Cl/F-TBIL Cl/F-LBW Cl2/F-BUN Tlag-adj CLcr

000000000000000000000000000100101000000000000001 X 7838.486 (7834.645 + 3.841) > 7835.122

cstep0160 V2/F-LDH Cl/F-TBIL Cl/F-LBW Cl2/F-BUN

000000000000000000100000000100101000000000000000 chosen, -2LL = 7829.178

Find effect to add that reduces -2LL the most

cstep0187 Ka-BUN V2/F-LDH Cl/F-TBIL Cl/F-LBW Cl2/F-BUN

100000000000000000100000000100101000000000000000 X 7829.766 (7825.925 + 3.841) > 7829.178

cstep0188 Ka-ALP V2/F-LDH Cl/F-TBIL Cl/F-LBW Cl2/F-BUN

010000000000000000100000000100101000000000000000 X 7832.954 (7829.113 + 3.841) > 7829.178

cstep0189 Ka-LDH V2/F-LDH Cl/F-TBIL Cl/F-LBW Cl2/F-BUN

001000000000000000100000000100101000000000000000 X 7832.986 (7829.144 + 3.841) > 7829.178

cstep0190 Ka-TBIL V2/F-LDH Cl/F-TBIL Cl/F-LBW Cl2/F-BUN

000100000000000000100000000100101000000000000000 X 7832.763 (7828.921 + 3.841) > 7829.178

cstep0191 Ka-AST V2/F-LDH Cl/F-TBIL Cl/F-LBW Cl2/F-BUN

000010000000000000100000000100101000000000000000 X 7832.650 (7828.809 + 3.841) > 7829.178

cstep0192 Ka-LBW V2/F-LDH Cl/F-TBIL Cl/F-LBW Cl2/F-BUN

000001000000000000100000000100101000000000000000 X 7831.639 (7827.798 + 3.841) > 7829.178

cstep0193 Ka-BMI V2/F-LDH Cl/F-TBIL Cl/F-LBW Cl2/F-BUN

000000100000000000100000000100101000000000000000 X 7831.771 (7827.930 + 3.841) > 7829.178

cstep0194 Ka-adj CLcr V2/F-LDH Cl/F-TBIL Cl/F-LBW Cl2/F-BUN

000000010000000000100000000100101000000000000000 X 7832.932 (7829.091 + 3.841) > 7829.178

cstep0195 V/F-BUN V2/F-LDH Cl/F-TBIL Cl/F-LBW Cl2/F-BUN

000000001000000000100000000100101000000000000000 X 7832.854 (7829.012 + 3.841) > 7829.178

cstep0196 V/F-ALP V2/F-LDH Cl/F-TBIL Cl/F-LBW Cl2/F-BUN

000000000100000000100000000100101000000000000000 X 7832.939 (7829.098 + 3.841) > 7829.178

cstep0197 V/F-LDH V2/F-LDH Cl/F-TBIL Cl/F-LBW Cl2/F-BUN

000000000010000000100000000100101000000000000000 X 7831.900 (7828.058 + 3.841) > 7829.178

cstep0198 V/F-TBIL V2/F-LDH Cl/F-TBIL Cl/F-LBW Cl2/F-BUN

000000000001000000100000000100101000000000000000 X 7832.829 (7828.987 + 3.841) > 7829.178

cstep0199 V/F-AST V2/F-LDH Cl/F-TBIL Cl/F-LBW Cl2/F-BUN

000000000000100000100000000100101000000000000000 X 7833.013 (7829.172 + 3.841) > 7829.178

cstep0200 V/F-BMI V2/F-LDH Cl/F-TBIL Cl/F-LBW Cl2/F-BUN

000000000000010000100000000100101000000000000000 X 7832.253 (7828.412 + 3.841) > 7829.178

cstep0201 V/F-LBW V2/F-LDH Cl/F-TBIL Cl/F-LBW Cl2/F-BUN

000000000000001000100000000100101000000000000000 X 7832.942 (7829.101 + 3.841) > 7829.178

cstep0202 V/F-adj CLcr V2/F-LDH Cl/F-TBIL Cl/F-LBW Cl2/F-BUN

000000000000000100100000000100101000000000000000 X 7832.432 (7828.590 + 3.841) > 7829.178

cstep0203 V2/F-BUN V2/F-LDH Cl/F-TBIL Cl/F-LBW Cl2/F-BUN

000000000000000010100000000100101000000000000000 X 7831.890 (7828.048 + 3.841) > 7829.178

cstep0204 V2/F-ALP V2/F-LDH Cl/F-TBIL Cl/F-LBW Cl2/F-BUN

000000000000000001100000000100101000000000000000 X 7832.900 (7829.059 + 3.841) > 7829.178

cstep0205 V2/F-LDH V2/F-TBIL Cl/F-TBIL Cl/F-LBW Cl2/F-BUN

000000000000000000110000000100101000000000000000 X 7833.629 (7829.787 + 3.841) > 7829.178

cstep0206 V2/F-LDH V2/F-AST Cl/F-TBIL Cl/F-LBW Cl2/F-BUN

000000000000000000101000000100101000000000000000 X 7832.709 (7828.868 + 3.841) > 7829.178

cstep0207 V2/F-LDH V2/F-BMI Cl/F-TBIL Cl/F-LBW Cl2/F-BUN

000000000000000000100100000100101000000000000000 X 7832.393 (7828.552 + 3.841) > 7829.178

cstep0208 V2/F-LDH V2/F-LBW Cl/F-TBIL Cl/F-LBW Cl2/F-BUN

000000000000000000100010000100101000000000000000 X 7832.573 (7828.732 + 3.841) > 7829.178

cstep0209 V2/F-LDH V2/F-adj CLcr Cl/F-TBIL Cl/F-LBW Cl2/F-BUN

000000000000000000100001000100101000000000000000 X 7832.611 (7828.769 + 3.841) > 7829.178

cstep0210 V2/F-LDH Cl/F-BUN Cl/F-TBIL Cl/F-LBW Cl2/F-BUN

000000000000000000100000100100101000000000000000 X 7832.824 (7828.983 + 3.841) > 7829.178

cstep0211 V2/F-LDH Cl/F-ALP Cl/F-TBIL Cl/F-LBW Cl2/F-BUN

000000000000000000100000010100101000000000000000 X 7832.068 (7828.226 + 3.841) > 7829.178

cstep0212 V2/F-LDH Cl/F-LDH Cl/F-TBIL Cl/F-LBW Cl2/F-BUN

000000000000000000100000001100101000000000000000 X 7832.604 (7828.762 + 3.841) > 7829.178

cstep0213 V2/F-LDH Cl/F-TBIL Cl/F-AST Cl/F-LBW Cl2/F-BUN

000000000000000000100000000110101000000000000000 X 7832.826 (7828.984 + 3.841) > 7829.178

cstep0214 V2/F-LDH Cl/F-TBIL Cl/F-BMI Cl/F-LBW Cl2/F-BUN

000000000000000000100000000101101000000000000000 X 7831.028 (7827.186 + 3.841) > 7829.178

cstep0215 V2/F-LDH Cl/F-TBIL Cl/F-LBW Cl/F-adj CLcr Cl2/F-BUN

000000000000000000100000000100111000000000000000 X 7833.006 (7829.164 + 3.841) > 7829.178

cstep0216 V2/F-LDH Cl/F-TBIL Cl/F-LBW Cl2/F-BUN Cl2/F-ALP

000000000000000000100000000100101100000000000000 X 7831.685 (7827.843 + 3.841) > 7829.178

cstep0217 V2/F-LDH Cl/F-TBIL Cl/F-LBW Cl2/F-BUN Cl2/F-LDH

000000000000000000100000000100101010000000000000 7826.780 (7822.938 + 3.841) < 7829.178

cstep0218 V2/F-LDH Cl/F-TBIL Cl/F-LBW Cl2/F-BUN Cl2/F-TBIL

000000000000000000100000000100101001000000000000 X 7832.870 (7829.029 + 3.841) > 7829.178

cstep0219 V2/F-LDH Cl/F-TBIL Cl/F-LBW Cl2/F-BUN Cl2/F-AST

000000000000000000100000000100101000100000000000 X 7832.034 (7828.193 + 3.841) > 7829.178

cstep0220 V2/F-LDH Cl/F-TBIL Cl/F-LBW Cl2/F-BUN Cl2/F-BMI

000000000000000000100000000100101000010000000000 X 7832.846 (7829.005 + 3.841) > 7829.178

cstep0221 V2/F-LDH Cl/F-TBIL Cl/F-LBW Cl2/F-BUN Cl2/F-LBW

000000000000000000100000000100101000001000000000 X 7832.313 (7828.471 + 3.841) > 7829.178

cstep0222 V2/F-LDH Cl/F-TBIL Cl/F-LBW Cl2/F-BUN Cl2/F-adj CLcr

000000000000000000100000000100101000000100000000 X 7846.067 (7842.226 + 3.841) > 7829.178

cstep0223 V2/F-LDH Cl/F-TBIL Cl/F-LBW Cl2/F-BUN Tlag-BUN

000000000000000000100000000100101000000010000000 X 7832.070 (7828.229 + 3.841) > 7829.178

cstep0224 V2/F-LDH Cl/F-TBIL Cl/F-LBW Cl2/F-BUN Tlag-ALP

000000000000000000100000000100101000000001000000 X 7832.979 (7829.137 + 3.841) > 7829.178

cstep0225 V2/F-LDH Cl/F-TBIL Cl/F-LBW Cl2/F-BUN Tlag-LDH

000000000000000000100000000100101000000000100000 X 7832.836 (7828.995 + 3.841) > 7829.178

cstep0226 V2/F-LDH Cl/F-TBIL Cl/F-LBW Cl2/F-BUN Tlag-TBIL

000000000000000000100000000100101000000000010000 X 7829.689 (7825.848 + 3.841) > 7829.178

cstep0227 V2/F-LDH Cl/F-TBIL Cl/F-LBW Cl2/F-BUN Tlag-AST

000000000000000000100000000100101000000000001000 X 7831.688 (7827.846 + 3.841) > 7829.178

cstep0228 V2/F-LDH Cl/F-TBIL Cl/F-LBW Cl2/F-BUN Tlag-BMI

000000000000000000100000000100101000000000000100 X 7831.021 (7827.179 + 3.841) > 7829.178

cstep0229 V2/F-LDH Cl/F-TBIL Cl/F-LBW Cl2/F-BUN Tlag-LBW

000000000000000000100000000100101000000000000010 X 7833.000 (7829.159 + 3.841) > 7829.178

cstep0230 V2/F-LDH Cl/F-TBIL Cl/F-LBW Cl2/F-BUN Tlag-adj CLcr

000000000000000000100000000100101000000000000001 X 7832.455 (7828.613 + 3.841) > 7829.178

cstep0217 V2/F-LDH Cl/F-TBIL Cl/F-LBW Cl2/F-BUN Cl2/F-LDH

000000000000000000100000000100101010000000000000 chosen, -2LL = 7822.938

Find effect to add that reduces -2LL the most

cstep0231 Ka-BUN V2/F-LDH Cl/F-TBIL Cl/F-LBW Cl2/F-BUN Cl2/F-LDH

100000000000000000100000000100101010000000000000 X 7823.434 (7819.593 + 3.841) > 7822.938

cstep0232 Ka-ALP V2/F-LDH Cl/F-TBIL Cl/F-LBW Cl2/F-BUN Cl2/F-LDH

010000000000000000100000000100101010000000000000 X 7826.702 (7822.861 + 3.841) > 7822.938

cstep0233 Ka-LDH V2/F-LDH Cl/F-TBIL Cl/F-LBW Cl2/F-BUN Cl2/F-LDH

001000000000000000100000000100101010000000000000 X 7826.518 (7822.676 + 3.841) > 7822.938

cstep0234 Ka-TBIL V2/F-LDH Cl/F-TBIL Cl/F-LBW Cl2/F-BUN Cl2/F-LDH

000100000000000000100000000100101010000000000000 X 7826.645 (7822.803 + 3.841) > 7822.938

cstep0235 Ka-AST V2/F-LDH Cl/F-TBIL Cl/F-LBW Cl2/F-BUN Cl2/F-LDH

000010000000000000100000000100101010000000000000 X 7825.832 (7821.990 + 3.841) > 7822.938

cstep0236 Ka-LBW V2/F-LDH Cl/F-TBIL Cl/F-LBW Cl2/F-BUN Cl2/F-LDH

000001000000000000100000000100101010000000000000 X 7825.268 (7821.426 + 3.841) > 7822.938

cstep0237 Ka-BMI V2/F-LDH Cl/F-TBIL Cl/F-LBW Cl2/F-BUN Cl2/F-LDH

000000100000000000010000000001001010100000000000000 X 7825.462 (7821.621 + 3.841) > 7822.938

cstep0238 Ka-adj CLcr V2/F-LDH Cl/F-TBIL Cl/F-LBW Cl2/F-BUN Cl2/F-LDH

000000010000000000010000000001001010100000000000000 X 7826.619 (7822.778 + 3.841) > 7822.938

cstep0239 V/F-BUN V2/F-LDH Cl/F-TBIL Cl/F-LBW Cl2/F-BUN Cl2/F-LDH

000000001000000000010000000001001010100000000000000 X 7826.677 (7822.836 + 3.841) > 7822.938

cstep0240 V/F-ALP V2/F-LDH Cl/F-TBIL Cl/F-LBW Cl2/F-BUN Cl2/F-LDH

000000000100000000010000000001001010100000000000000 X 7826.599 (7822.758 + 3.841) > 7822.938

cstep0241 V/F-LDH V2/F-LDH Cl/F-TBIL Cl/F-LBW Cl2/F-BUN Cl2/F-LDH

000000000010000000010000000001001010100000000000000 X 7826.126 (7822.284 + 3.841) > 7822.938

cstep0242 V/F-TBIL V2/F-LDH Cl/F-TBIL Cl/F-LBW Cl2/F-BUN Cl2/F-LDH

000000000001000000010000000001001010100000000000000 X 7826.534 (7822.693 + 3.841) > 7822.938

cstep0243 V/F-AST V2/F-LDH Cl/F-TBIL Cl/F-LBW Cl2/F-BUN Cl2/F-LDH

000000000000100000010000000001001010100000000000000 X 7826.726 (7822.885 + 3.841) > 7822.938

cstep0244 V/F-BMI V2/F-LDH Cl/F-TBIL Cl/F-LBW Cl2/F-BUN Cl2/F-LDH

000000000000010000010000000001001010100000000000000 X 7825.496 (7821.655 + 3.841) > 7822.938

cstep0245 V/F-LBW V2/F-LDH Cl/F-TBIL Cl/F-LBW Cl2/F-BUN Cl2/F-LDH

000000000000001000010000000001001010100000000000000 X 7826.687 (7822.846 + 3.841) > 7822.938

cstep0246 V/F-adj CLcr V2/F-LDH Cl/F-TBIL Cl/F-LBW Cl2/F-BUN Cl2/F-LDH

000000000000000100010000000001001010100000000000000 X 7826.201 (7822.360 + 3.841) > 7822.938

cstep0247 V2/F-BUN V2/F-LDH Cl/F-TBIL Cl/F-LBW Cl2/F-BUN Cl2/F-LDH

000000000000000010010000000001001010100000000000000 X 7825.170 (7821.329 + 3.841) > 7822.938

cstep0248 V2/F-ALP V2/F-LDH Cl/F-TBIL Cl/F-LBW Cl2/F-BUN Cl2/F-LDH

000000000000000001010000000001001010100000000000000 X 7826.682 (7822.840 + 3.841) > 7822.938

cstep0249 V2/F-LDH V2/F-TBIL Cl/F-TBIL Cl/F-LBW Cl2/F-BUN Cl2/F-LDH

000000000000000000011000000001001010100000000000000 X 7826.715 (7822.874 + 3.841) > 7822.938

cstep0250 V2/F-LDH V2/F-AST Cl/F-TBIL Cl/F-LBW Cl2/F-BUN Cl2/F-LDH

000000000000000000010100000001001010100000000000000 X 7826.076 (7822.234 + 3.841) > 7822.938

cstep0251 V2/F-LDH V2/F-BMI Cl/F-TBIL Cl/F-LBW Cl2/F-BUN Cl2/F-LDH

000000000000000000010010000001001010100000000000000 X 7825.994 (7822.153 + 3.841) > 7822.938

cstep0252 V2/F-LDH V2/F-LBW Cl/F-TBIL Cl/F-LBW Cl2/F-BUN Cl2/F-LDH

000000000000000000010001000001001010100000000000000 X 7826.346 (7822.505 + 3.841) > 7822.938

cstep0253 V2/F-LDH V2/F-adj CLcr Cl/F-TBIL Cl/F-LBW Cl2/F-BUN Cl2/F-LDH

000000000000000000010000100001001010100000000000000 X 7826.230 (7822.389 + 3.841) > 7822.938

cstep0254 V2/F-LDH Cl/F-BUN Cl/F-TBIL Cl/F-LBW Cl2/F-BUN Cl2/F-LDH

000000000000000000010000010001001010100000000000000 X 7826.336 (7822.495 + 3.841) > 7822.938

cstep0255 V2/F-LDH Cl/F-ALP Cl/F-TBIL Cl/F-LBW Cl2/F-BUN Cl2/F-LDH

000000000000000000010000001001001010100000000000000 X 7825.788 (7821.947 + 3.841) > 7822.938

cstep0256 V2/F-LDH Cl/F-LDH Cl/F-TBIL Cl/F-LBW Cl2/F-BUN Cl2/F-LDH

000000000000000000010000000101001010100000000000000 X 7824.260 (7820.418 + 3.841) > 7822.938

cstep0257 V2/F-LDH Cl/F-TBIL Cl/F-AST Cl/F-LBW Cl2/F-BUN Cl2/F-LDH

000000000000000000010000000001101010100000000000000 X 7825.545 (7821.703 + 3.841) > 7822.938

cstep0258 V2/F-LDH Cl/F-TBIL Cl/F-BMI Cl/F-LBW Cl2/F-BUN Cl2/F-LDH

000000000000000000010000000001011010100000000000000 X 7825.222 (7821.381 + 3.841) > 7822.938

cstep0259 V2/F-LDH Cl/F-TBIL Cl/F-LBW Cl/F-adj CLcr Cl2/F-BUN Cl2/F-LDH

000000000000000000100000000100111010000000000000 X 7826.870 (7823.028 + 3.841) > 7822.938

cstep0260 V2/F-LDH Cl/F-TBIL Cl/F-LBW Cl2/F-BUN Cl2/F-ALP Cl2/F-LDH

000000000000000000100000000100101110000000000000 X 7835.070 (7831.229 + 3.841) > 7822.938

cstep0261 V2/F-LDH Cl/F-TBIL Cl/F-LBW Cl2/F-BUN Cl2/F-LDH Cl2/F-TBIL

000000000000000000100000000100101011000000000000 X 7825.175 (7821.334 + 3.841) > 7822.938

cstep0262 V2/F-LDH Cl/F-TBIL Cl/F-LBW Cl2/F-BUN Cl2/F-LDH Cl2/F-AST

000000000000000000100000000100101010100000000000 X 7826.584 (7822.742 + 3.841) > 7822.938

cstep0263 V2/F-LDH Cl/F-TBIL Cl/F-LBW Cl2/F-BUN Cl2/F-LDH Cl2/F-BMI

000000000000000000100000000100101010010000000000 X 7826.628 (7822.787 + 3.841) > 7822.938

cstep0264 V2/F-LDH Cl/F-TBIL Cl/F-LBW Cl2/F-BUN Cl2/F-LDH Cl2/F-LBW

000000000000000000100000000100101010001000000000 7822.226 (7818.384 + 3.841) < 7822.938

cstep0265 V2/F-LDH Cl/F-TBIL Cl/F-LBW Cl2/F-BUN Cl2/F-LDH Cl2/F-adj CLcr

000000000000000000100000000100101010000100000000 X 7826.834 (7822.993 + 3.841) > 7822.938

cstep0266 V2/F-LDH Cl/F-TBIL Cl/F-LBW Cl2/F-BUN Cl2/F-LDH Tlag-BUN

000000000000000000100000000100101010000010000000 X 7826.123 (7822.281 + 3.841) > 7822.938

cstep0267 V2/F-LDH Cl/F-TBIL Cl/F-LBW Cl2/F-BUN Cl2/F-LDH Tlag-ALP

000000000000000000100000000100101010000001000000 X 7826.996 (7823.154 + 3.841) > 7822.938

cstep0268 V2/F-LDH Cl/F-TBIL Cl/F-LBW Cl2/F-BUN Cl2/F-LDH Tlag-LDH

000000000000000000100000000100101010000000100000 X 7826.519 (7822.677 + 3.841) > 7822.938

cstep0269 V2/F-LDH Cl/F-TBIL Cl/F-LBW Cl2/F-BUN Cl2/F-LDH Tlag-TBIL

000000000000000000100000000100101010000000010000 X 7823.497 (7819.656 + 3.841) > 7822.938

cstep0270 V2/F-LDH Cl/F-TBIL Cl/F-LBW Cl2/F-BUN Cl2/F-LDH Tlag-AST

000000000000000000100000000100101010000000001000 X 7825.351 (7821.510 + 3.841) > 7822.938

cstep0271 V2/F-LDH Cl/F-TBIL Cl/F-LBW Cl2/F-BUN Cl2/F-LDH Tlag-BMI

000000000000000000100000000100101010000000000100 X 7823.021 (7819.180 + 3.841) > 7822.938

cstep0272 V2/F-LDH Cl/F-TBIL Cl/F-LBW Cl2/F-BUN Cl2/F-LDH Tlag-LBW

000000000000000000100000000100101010000000000010 X 7826.636 (7822.794 + 3.841) > 7822.938

cstep0273 V2/F-LDH Cl/F-TBIL Cl/F-LBW Cl2/F-BUN Cl2/F-LDH Tlag-adj CLcr

000000000000000000100000000100101010000000000001 X 7826.255 (7822.414 + 3.841) > 7822.938

cstep0264 V2/F-LDH Cl/F-TBIL Cl/F-LBW Cl2/F-BUN Cl2/F-LDH Cl2/F-LBW

000000000000000000100000000100101010001000000000 chosen, -2LL = 7818.384

Find effect to add that reduces -2LL the most

cstep0274 Ka-BUN V2/F-LDH Cl/F-TBIL Cl/F-LBW Cl2/F-BUN Cl2/F-LDH Cl2/F-LBW

100000000000000000100000000100101010001000000000 X 7819.046 (7815.205 + 3.841) > 7818.384

cstep0275 Ka-ALP V2/F-LDH Cl/F-TBIL Cl/F-LBW Cl2/F-BUN Cl2/F-LDH Cl2/F-LBW

010000000000000000100000000100101010001000000000 X 7822.244 (7818.402 + 3.841) > 7818.384

cstep0276 Ka-LDH V2/F-LDH Cl/F-TBIL Cl/F-LBW Cl2/F-BUN Cl2/F-LDH Cl2/F-LBW

001000000000000000100000000100101010001000000000 X 7821.980 (7818.139 + 3.841) > 7818.384

cstep0277 Ka-TBIL V2/F-LDH Cl/F-TBIL Cl/F-LBW Cl2/F-BUN Cl2/F-LDH Cl2/F-LBW

000100000000000000100000000100101010001000000000 X 7821.766 (7817.925 + 3.841) > 7818.384

cstep0278 Ka-AST V2/F-LDH Cl/F-TBIL Cl/F-LBW Cl2/F-BUN Cl2/F-LDH Cl2/F-LBW

000010000000000000100000000100101010001000000000 X 7821.399 (7817.558 + 3.841) > 7818.384

cstep0279 Ka-LBW V2/F-LDH Cl/F-TBIL Cl/F-LBW Cl2/F-BUN Cl2/F-LDH Cl2/F-LBW

000001000000000000100000000100101010001000000000 X 7821.180 (7817.338 + 3.841) > 7818.384

cstep0280 Ka-BMI V2/F-LDH Cl/F-TBIL Cl/F-LBW Cl2/F-BUN Cl2/F-LDH Cl2/F-LBW

000000100000000000100000000100101010001000000000 X 7820.978 (7817.136 + 3.841) > 7818.384

cstep0281 Ka-adj CLcr V2/F-LDH Cl/F-TBIL Cl/F-LBW Cl2/F-BUN Cl2/F-LDH Cl2/F-LBW

000000010000000000100000000100101010001000000000 X 7821.911 (7818.069 + 3.841) > 7818.384

cstep0282 V/F-BUN V2/F-LDH Cl/F-TBIL Cl/F-LBW Cl2/F-BUN Cl2/F-LDH Cl2/F-LBW

000000001000000000100000000100101010001000000000 X 7822.288 (7818.447 + 3.841) > 7818.384

cstep0283 V/F-ALP V2/F-LDH Cl/F-TBIL Cl/F-LBW Cl2/F-BUN Cl2/F-LDH Cl2/F-LBW

000000000100000000100000000100101010001000000000 X 7822.110 (7818.269 + 3.841) > 7818.384

cstep0284 V/F-LDH V2/F-LDH Cl/F-TBIL Cl/F-LBW Cl2/F-BUN Cl2/F-LDH Cl2/F-LBW

000000000010000000100000000100101010001000000000 X 7821.890 (7818.049 + 3.841) > 7818.384

cstep0285 V/F-TBIL V2/F-LDH Cl/F-TBIL Cl/F-LBW Cl2/F-BUN Cl2/F-LDH Cl2/F-LBW

000000000001000000100000000100101010001000000000 X 7821.987 (7818.146 + 3.841) > 7818.384

cstep0286 V/F-AST V2/F-LDH Cl/F-TBIL Cl/F-LBW Cl2/F-BUN Cl2/F-LDH Cl2/F-LBW

000000000000100000100000000100101010001000000000 X 7822.213 (7818.371 + 3.841) > 7818.384

cstep0287 V/F-BMI V2/F-LDH Cl/F-TBIL Cl/F-LBW Cl2/F-BUN Cl2/F-LDH Cl2/F-LBW

000000000000010000100000000100101010001000000000 X 7821.119 (7817.277 + 3.841) > 7818.384

cstep0288 V/F-LBW V2/F-LDH Cl/F-TBIL Cl/F-LBW Cl2/F-BUN Cl2/F-LDH Cl2/F-LBW

000000000000001000100000000100101010001000000000 X 7821.226 (7817.385 + 3.841) > 7818.384

cstep0289 V/F-adj CLcr V2/F-LDH Cl/F-TBIL Cl/F-LBW Cl2/F-BUN Cl2/F-LDH Cl2/F-LBW

000000000000000100100000000100101010001000000000 X 7821.029 (7817.187 + 3.841) > 7818.384

cstep0290 V2/F-BUN V2/F-LDH Cl/F-TBIL Cl/F-LBW Cl2/F-BUN Cl2/F-LDH Cl2/F-LBW

000000000000000010100000000100101010001000000000 X 7822.025 (7818.183 + 3.841) > 7818.384

cstep0291 V2/F-ALP V2/F-LDH Cl/F-TBIL Cl/F-LBW Cl2/F-BUN Cl2/F-LDH Cl2/F-LBW

000000000000000001100000000100101010001000000000 X 7821.344 (7817.502 + 3.841) > 7818.384

cstep0292 V2/F-LDH V2/F-TBIL Cl/F-TBIL Cl/F-LBW Cl2/F-BUN Cl2/F-LDH Cl2/F-LBW

000000000000000000110000000100101010001000000000 X 7821.555 (7817.714 + 3.841) > 7818.384

cstep0293 V2/F-LDH V2/F-AST Cl/F-TBIL Cl/F-LBW Cl2/F-BUN Cl2/F-LDH Cl2/F-LBW

000000000000000000101000000100101010001000000000 X 7821.342 (7817.500 + 3.841) > 7818.384

cstep0294 V2/F-LDH V2/F-BMI Cl/F-TBIL Cl/F-LBW Cl2/F-BUN Cl2/F-LDH Cl2/F-LBW

000000000000000000100100000100101010001000000000 X 7822.598 (7818.757 + 3.841) > 7818.384

cstep0295 V2/F-LDH V2/F-LBW Cl/F-TBIL Cl/F-LBW Cl2/F-BUN Cl2/F-LDH Cl2/F-LBW

000000000000000000100010000100101010001000000000 X 7822.028 (7818.187 + 3.841) > 7818.384

cstep0296 V2/F-LDH V2/F-adj CLcr Cl/F-TBIL Cl/F-LBW Cl2/F-BUN Cl2/F-LDH Cl2/F-LBW

000000000000000000100001000100101010001000000000 X 7821.589 (7817.747 + 3.841) > 7818.384

cstep0297 V2/F-LDH Cl/F-BUN Cl/F-TBIL Cl/F-LBW Cl2/F-BUN Cl2/F-LDH Cl2/F-LBW

000000000000000000100000100100101010001000000000 X 7821.944 (7818.102 + 3.841) > 7818.384

cstep0298 V2/F-LDH Cl/F-ALP Cl/F-TBIL Cl/F-LBW Cl2/F-BUN Cl2/F-LDH Cl2/F-LBW

000000000000000000100000010100101010001000000000 X 7821.781 (7817.940 + 3.841) > 7818.384

cstep0299 V2/F-LDH Cl/F-LDH Cl/F-TBIL Cl/F-LBW Cl2/F-BUN Cl2/F-LDH Cl2/F-LBW

000000000000000000100000001100101010001000000000 X 7820.758 (7816.916 + 3.841) > 7818.384

cstep0300 V2/F-LDH Cl/F-TBIL Cl/F-AST Cl/F-LBW Cl2/F-BUN Cl2/F-LDH Cl2/F-LBW

000000000000000000100000000110101010001000000000 X 7821.519 (7817.678 + 3.841) > 7818.384

cstep0301 V2/F-LDH Cl/F-TBIL Cl/F-BMI Cl/F-LBW Cl2/F-BUN Cl2/F-LDH Cl2/F-LBW

000000000000000000100000000101101010001000000000 X 7820.126 (7816.285 + 3.841) > 7818.384

cstep0302 V2/F-LDH Cl/F-TBIL Cl/F-LBW Cl/F-adj CLcr Cl2/F-BUN Cl2/F-LDH Cl2/F-LBW

000000000000000000100000000100111010001000000000 X 7830.869 (7827.027 + 3.841) > 7818.384

cstep0303 V2/F-LDH Cl/F-TBIL Cl/F-LBW Cl2/F-BUN Cl2/F-ALP Cl2/F-LDH Cl2/F-LBW

000000000000000000100000000100101110001000000000 X 7820.904 (7817.063 + 3.841) > 7818.384

cstep0304 V2/F-LDH Cl/F-TBIL Cl/F-LBW Cl2/F-BUN Cl2/F-LDH Cl2/F-TBIL Cl2/F-LBW

000000000000000000100000000100101011001000000000 X 7818.820 (7814.978 + 3.841) > 7818.384

cstep0305 V2/F-LDH Cl/F-TBIL Cl/F-LBW Cl2/F-BUN Cl2/F-LDH Cl2/F-AST Cl2/F-LBW

000000000000000000100000000100101010101000000000 X 7822.228 (7818.387 + 3.841) > 7818.384

cstep0306 V2/F-LDH Cl/F-TBIL Cl/F-LBW Cl2/F-BUN Cl2/F-LDH Cl2/F-BMI Cl2/F-LBW

000000000000000000100000000100101010011000000000 X 7821.838 (7817.996 + 3.841) > 7818.384

cstep0307 V2/F-LDH Cl/F-TBIL Cl/F-LBW Cl2/F-BUN Cl2/F-LDH Cl2/F-LBW Cl2/F-adj CLcr

000000000000000000100000000100101010001100000000 X 7822.380 (7818.538 + 3.841) > 7818.384

cstep0308 V2/F-LDH Cl/F-TBIL Cl/F-LBW Cl2/F-BUN Cl2/F-LDH Cl2/F-LBW Tlag-BUN

000000000000000000100000000100101010001010000000 X 7821.270 (7817.428 + 3.841) > 7818.384

cstep0309 V2/F-LDH Cl/F-TBIL Cl/F-LBW Cl2/F-BUN Cl2/F-LDH Cl2/F-LBW Tlag-ALP

000000000000000000100000000100101010001001000000 X 7823.854 (7820.012 + 3.841) > 7818.384

cstep0310 V2/F-LDH Cl/F-TBIL Cl/F-LBW Cl2/F-BUN Cl2/F-LDH Cl2/F-LBW Tlag-LDH

000000000000000000100000000100101010001000100000 X 7822.035 (7818.193 + 3.841) > 7818.384

cstep0311 V2/F-LDH Cl/F-TBIL Cl/F-LBW Cl2/F-BUN Cl2/F-LDH Cl2/F-LBW Tlag-TBIL

000000000000000000100000000100101010001000010000 X 7821.089 (7817.248 + 3.841) > 7818.384

cstep0312 V2/F-LDH Cl/F-TBIL Cl/F-LBW Cl2/F-BUN Cl2/F-LDH Cl2/F-LBW Tlag-AST

000000000000000000100000000100101010001000001000 X 7820.869 (7817.027 + 3.841) > 7818.384

cstep0313 V2/F-LDH Cl/F-TBIL Cl/F-LBW Cl2/F-BUN Cl2/F-LDH Cl2/F-LBW Tlag-BMI

000000000000000000100000000100101010001000000100 X 7818.594 (7814.753 + 3.841) > 7818.384

cstep0314 V2/F-LDH Cl/F-TBIL Cl/F-LBW Cl2/F-BUN Cl2/F-LDH Cl2/F-LBW Tlag-LBW

000000000000000000100000000100101010001000000010 X 7822.139 (7818.297 + 3.841) > 7818.384

cstep0315 V2/F-LDH Cl/F-TBIL Cl/F-LBW Cl2/F-BUN Cl2/F-LDH Cl2/F-LBW Tlag-adj CLcr

000000000000000000100000000100101010001000000001 X 7821.797 (7817.956 + 3.841) > 7818.384

No effect chosen to add

Find effect to subtract that increases -2LL the least

cstep0316 Cl/F-TBIL Cl/F-LBW Cl2/F-BUN Cl2/F-LDH Cl2/F-LBW

000000000000000000000000000100101010001000000000 X 7825.008 (7831.642 - 6.635) > 7818.384

cstep0317 V2/F-LDH Cl/F-LBW Cl2/F-BUN Cl2/F-LDH Cl2/F-LBW

000000000000000000100000000000101010001000000000 X 7820.902 (7827.537 - 6.635) > 7818.384

cstep0318 V2/F-LDH Cl/F-TBIL Cl2/F-BUN Cl2/F-LDH Cl2/F-LBW

000000000000000000100000000100001010001000000000 X 7820.759 (7827.394 - 6.635) > 7818.384

cstep0319 V2/F-LDH Cl/F-TBIL Cl/F-LBW Cl2/F-LDH Cl2/F-LBW

000000000000000000100000000100100010001000000000 7815.935 (7822.570 - 6.635) < 7818.384

cstep0221 V2/F-LDH Cl/F-TBIL Cl/F-LBW Cl2/F-BUN Cl2/F-LBW

00000000000000000010000000010010100000010000000000 X 7821.836 (7828.471 - 6.635) > 7818.384

cstep0217 V2/F-LDH Cl/F-TBIL Cl/F-LBW Cl2/F-BUN Cl2/F-LDH

00000000000000000010000000010010101000000000000000 7816.304 (7822.938 - 6.635) < 7818.384

cstep0319 V2/F-LDH Cl/F-TBIL Cl/F-LBW Cl2/F-LDH Cl2/F-LBW

00000000000000000010000000010010001000100000000000 chosen, 7822.570

Find effect to subtract that increases -2LL the least

cstep0320 Cl/F-TBIL Cl/F-LBW Cl2/F-LDH Cl2/F-LBW

00000000000000000000000000010010001000100000000000 X 7828.596 (7835.231 - 6.635) > 7822.570

cstep0321 V2/F-LDH Cl/F-LBW Cl2/F-LDH Cl2/F-LBW

00000000000000000010000000000010001000100000000000 X 7825.470 (7832.105 - 6.635) > 7822.570

cstep0322 V2/F-LDH Cl/F-TBIL Cl2/F-LDH Cl2/F-LBW

00000000000000000010000000010000001000100000000000 X 7838.358 (7844.993 - 6.635) > 7822.570

cstep0323 V2/F-LDH Cl/F-TBIL Cl/F-LBW Cl2/F-LBW

00000000000000000010000000010010000000100000000000 7821.812 (7828.447 - 6.635) < 7822.570

cstep0324 V2/F-LDH Cl/F-TBIL Cl/F-LBW Cl2/F-LDH

00000000000000000010000000010010001000000000000000 X 7832.235 (7838.870 - 6.635) > 7822.570

cstep0323 V2/F-LDH Cl/F-TBIL Cl/F-LBW Cl2/F-LBW

00000000000000000010000000010010000000100000000000 chosen, 7828.447

Find effect to subtract that increases -2LL the least

cstep0325 Cl/F-TBIL Cl/F-LBW Cl2/F-LBW 00000000000000000000000000010010000000100000000000 X 7829.948 (7836.583 - 6.635) > 7828.447

cstep0326 V2/F-LDH Cl/F-LBW Cl2/F-LBW 00000000000000000010000000000010000000100000000000 X 7837.042 (7843.677 - 6.635) > 7828.447

cstep0327 V2/F-LDH Cl/F-TBIL Cl2/F-LBW 00000000000000000010000000010000000000100000000000 X 7830.231 (7836.866 - 6.635) > 7828.447

cstep0328 V2/F-LDH Cl/F-TBIL Cl/F-LBW 00000000000000000010000000010010000000000000000000 X 7832.800 (7839.435 - 6.635) > 7828.447

No effect chosen to subtract

Scenario to use = cstep0323 V2/F-LDH Cl/F-TBIL Cl/F-LBW Cl2/F-LBW 00000000000000000010000000010010000000100000000000

Explanation of Stepwise Text: In the process of stepwise screening of covariates, the software's built-in program considers incorporating covariates one by one in order and then selecting the optimal combination for the next round of covariate screening. During the establishment stage of the covariate model, the effects of covariates BUN, ALP, LDH, TBIL, AST, BMI, LBW and adj CLcr were tested on model parameters, including K_a, T_{lag}, V/F, V_2/F, CL/F and CL_2/F. The final covariate model is the optimal result: CL/F-TBIL-LBW CL_2/F-LBW V_2/F-LDH.
